# Cytoplasmic TAF2–TAF8–TAF10 complex provides evidence for nuclear holo–TFIID assembly from preformed submodules

**DOI:** 10.1038/ncomms7011

**Published:** 2015-01-14

**Authors:** Simon Trowitzsch, Cristina Viola, Elisabeth Scheer, Sascha Conic, Virginie Chavant, Marjorie Fournier, Gabor Papai, Ima-Obong Ebong, Christiane Schaffitzel, Juan Zou, Matthias Haffke, Juri Rappsilber, Carol V. Robinson, Patrick Schultz, Laszlo Tora, Imre Berger

**Affiliations:** 1 European Molecular Biology Laboratory, Grenoble Outstation, 6 rue Jules Horowitz, 38042 Grenoble, France; 2 Unit for Virus Host-Cell Interactions, University Grenoble Alpes-EMBL-CNRS, 6 rue Jules Horowitz, 38042 Grenoble, France; 3 Cellular Signaling and Nuclear Dynamics Program, Institut de Génétique et de Biologie Moléculaire et Cellulaire, UMR 7104, INSERM U964, 1 rue Laurent Fries, 67404 Illkirch, France; 4 Proteomics Platform, Institut de Génétique et de Biologie Moléculaire et Cellulaire, UMR 7104, INSERM U964, 1 rue Laurent Fries, 67404 Illkirch, France; 5 Integrated Structural Biology Department, Institut de Génétique et de Biologie Moléculaire et Cellulaire, UMR 7104, INSERM U964, 1 rue Laurent Fries, 67404 Illkirch, France; 6 Chemistry Research Laboratory, University of Oxford, South Parks Road, Oxford OX1 3TA, UK; 7 Wellcome Trust Centre for Cell Biology, University of Edinburgh, Mayfield Road, Edinburgh EH9 3JR, UK; 8 Institute of Bioanalytics, Department of Biotechnology, Technische Universität Berlin, 13353 Berlin, Germany; 9 School of Biochemistry, Bristol University, Bristol BS8 1TD, UK

## Abstract

General transcription factor TFIID is a cornerstone of RNA polymerase II transcription initiation in eukaryotic cells. How human TFIID—a megadalton-sized multiprotein complex composed of the TATA-binding protein (TBP) and 13 TBP-associated factors (TAFs)—assembles into a functional transcription factor is poorly understood. Here we describe a heterotrimeric TFIID subcomplex consisting of the TAF2, TAF8 and TAF10 proteins, which assembles in the cytoplasm. Using native mass spectrometry, we define the interactions between the TAFs and uncover a central role for TAF8 in nucleating the complex. X-ray crystallography reveals a non-canonical arrangement of the TAF8–TAF10 histone fold domains. TAF2 binds to multiple motifs within the TAF8 C-terminal region, and these interactions dictate TAF2 incorporation into a core–TFIID complex that exists in the nucleus. Our results provide evidence for a stepwise assembly pathway of nuclear holo–TFIID, regulated by nuclear import of preformed cytoplasmic submodules.

## Abstract (web summary)

TFIID is an essential transcription factor complex that controls the expression of most protein-coding genes in eukaryotes. Here the authors identify and characterize a complex containing TAF2, TAF8 and TAF10, which assembles in the cytoplasm before integration into the nuclear holo–TFIID complex.

## Graphical abstract

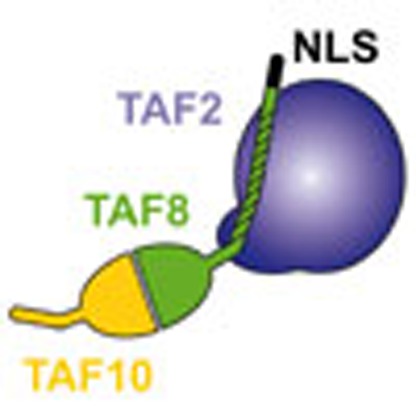

Eukaryotic class II gene transcription is controlled by a plethora of proteins, which are preassembled in large multiprotein complexes, including RNA polymerase II, Mediator and the general transcription factors (GTFs)^1^. The sequential nucleation of GTFs and Mediator on core promoter DNA initiates regulated class II gene transcription^2^. The GTF TFIID plays a central role in this process by linking cellular signalling events with regulatory DNA elements and the transcription machinery^3^. Although a basal transcription system supporting initiation of transcription from TATA-box-containing promoters can be reconstituted with TATA-binding protein (TBP), TFIIA, TFIIB, TFIIE, TFIIF and TFIIH *in vitro*, TFIID is additionally required to respond to activators and for efficient transcription from TATA-less promoters^4^,^5^. In mammalian cells, most of the expressed protein-coding gene promoters are occupied by TFIID and loss of TFIID components leads to embryonic lethality^6^,^7^,^8^,^9^. TFIID subunits are implicated in crosstalk with epigenetic modifications on nucleosomes and regulatory DNA elements in promoter regions^10^,^11^. Structural analysis of TFIID by cryo-electron microscopy revealed the overall architecture of TFIID and provided important insights into subunit assembly and promoter recognition at low to medium resolution^12^,^13^,^14^,^15^,^16^.

Canonical human TFIID consists of TBP and 13 TBP-associated factors (TAFs)^17^. Other non-canonical TFIID and TAF-containing complexes have been identified recently with key roles during spermatogenesis and stem cell development^18^,^19^,^20^. A central scaffold of canonical TFIID comprises two copies each of TAF4, 5, 6, 9 and 12, which were shown to form a symmetric core^12^,^21^. This core–TFIID complex was first identified in *Drosophila melanogaster* nuclei^21^. TAF3, 4, 6, 8, 9, 10, 11, 12 and 13 contain histone fold domains (HFDs), which stabilize discrete heterodimers (TAF3–10, TAF4–12, TAF6–9, TAF8–10 and TAF11–13) (refs 22, 23, 24, 25). Among these HFD pairs, the TAF8–10 heterodimer plays a key role in the TFIID assembly pathway, is critical for the integrity of holo–TFIID and also fulfills essential functions in early embryonic development^6^,^8^,^26^,^27^. Binding of TAF8–10 to core–TFIID triggers a transition from symmetry to asymmetry, which was proposed to prime the recruitment of TAF1, 2, 3, 7, 11, 13 and TBP to complete holo–TFIID^12^.

Evidence from genetic and biochemical studies showed that knockout of the TAF10 gene leads to impairment of mature TFIID assembly in F9 EC cells and to dissociation of TFIID in hepatocytes^6^,^26^,^27^. Biochemical data suggested that TAF8 and TAF10 interact strongly and specifically with each other via their HFDs^28^. Identification of human TAF8 uncovered high sequence similarities with the *Drosophila* protein PRODOS and the mouse TBN protein^8^,^28^,^29^. Mouse embryos carrying a mutation in TBN develop normally to the blastocyst stage but fail to develop further due to the lack of inner cell mass cells^8^. Interestingly, the same phenotype was also found in TAF10-knockout mice strongly suggesting that TAF8 and TAF10 are both involved in controlling embryonic development at similar stages^6^. The importance of this cooperative activity of TAF8 and TAF10 is supported by nuclear import assays, which showed that the transport of TAF10 from the cytoplasm to the nucleus depends on the nuclear localization signal (NLS) found at the carboxyl-terminal (C-terminal) end of TAF8 (ref. 30).

Human TAF2 (originally called either CIF150 or TAF_II_150) has been previously described as an essential cofactor for TFIID-dependent transcription from promoters with initiator (Inr)-containing promoter elements^31^,^32^,^33^. Later it was suggested that a trimeric TBP–TAF1–TAF2 complex is minimally required for efficient utilization of the Inr and downstream promoter elements^11^. TFIID complexes containing or lacking TAF2 have been described^31^,^34^ further suggesting that different types of TFIID complexes may exist in human cell nuclei. Recently, mutations in the TAF2-coding gene were shown to be associated with various neurological disorders^35^,^36^. Human TAF2 is predicted to adopt an aminopeptidase-like fold with an additional C-terminal unstructured region. Localization studies using immunopurified TFIID showed that TAF2 is an integral part of the central lobe of the holo-complex^13^.

While general functions of individual TFIID subunits and the holo-complex are increasingly better understood, very little is known to date about how the cell assembles this essential multiprotein complex. The existence of physiological core–TFIID in the nucleus, containing a subset of TAFs, provides evidence that the holo-complex may be assembled in a regulated manner from stable, preformed partial TFIID subassemblies. The dependence of some of the TAFs on each other for nuclear import and the critical role of the TAF8–10 pair in functional remodelling of core–TFIID imply that discrete submodules preassemble also in the cytoplasm of cells. However, direct evidence for the presence of subassemblies in the cytoplasm is lacking to date.

By immunoprecipitating TAF-containing complexes from different cellular compartments, we identify a novel endogenous TFIID subcomplex formed by TAF2, 8 and 10 in the cytoplasm of human cells. We dissect cytoplasmic TAF2–8–10 biochemically and structurally. We elucidate the interactions that stabilize the complex and reveal a central role of TAF8 in its nucleation. By X-ray crystallography, we demonstrate a non-canonical histone-fold domain pair arrangement between TAF8 and TAF10. We report a novel interaction between TAF8 and TAF2, mediated by multiple peptide motifs in the TAF8 C-terminal region. Moreover, we describe the formation of a putative nuclear import particle comprising the TAF2–8–10 complex and Importin α1. Furthermore, we demonstrate that the TAF2–TAF8 interaction is not only crucial for formation of the cytoplasmic TAF2–8–10 complex, but also dictates incorporation of TAF2 into a physiological core–TFIID complex that exists in the nucleus.

## Results

### An endogenous cytoplasmic TAF2–8–10 complex

**Figure 1:**
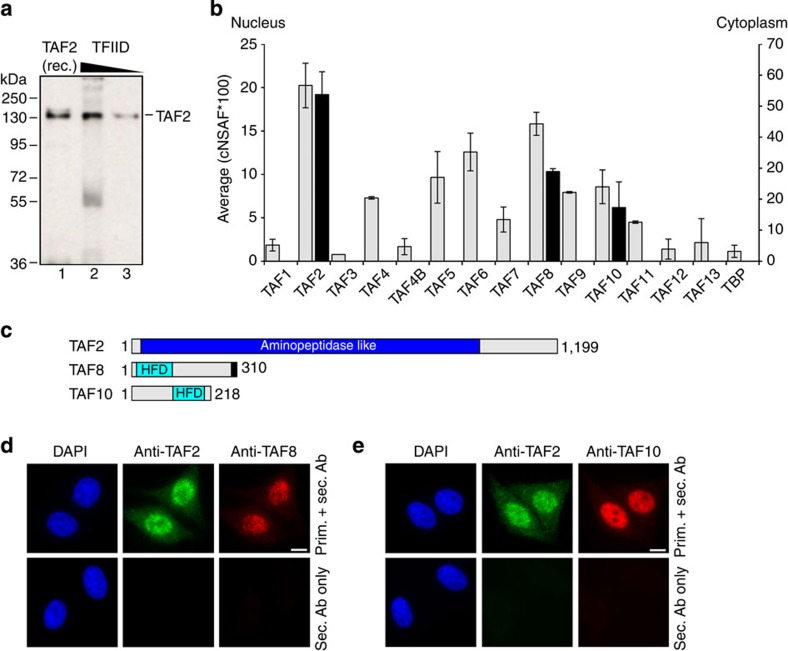
A TAF2–8–10 complex exists in the cytoplasm. (**a**) Purified, polyclonal anti-TAF2 antibodies specifically recognize recombinant and endogenous TAF2. Recombinant (rec.) purified TAF2 (10 ng, lane 1) and immunopurified TFIID (300 and 150 ng; lanes 2, 3) were loaded on an 8% SDS–PAGE, blotted and analysed by western blot assay. Protein size markers are indicated. (**b**) Abundances of individual proteins co-immunoprecipitated from nuclear or cytoplasmic HeLa cell extracts (grey or black bars, respectively) using purified polyclonal anti-TAF2 antibodies were compared in units of normalized spectral abundance factors (NSAFs). Each column is the average of two independent experiments and error bars represent range of the data. (**c**) Domain organization of TAF2, TAF8 and TAF10 in a schematic view. Grey rectangles indicate predicted, unstructured regions. The NLS of TAF8 is shown as a black bar. Numbers indicate first and last amino acids in each protein. (**d**) Immunofluorescence microscopy of HeLa cells. Nuclei are visualized by 4′,6-diamidino-2-phenylindole (DAPI) staining (blue). TAF2 is displayed in green and TAF8 in red. The bottom panel shows images of control cells, which were treated with secondary antibodies only. Scale bar, 10 μm. (**e**) Immunofluorescence microscopy of HeLa cells as in **d**, but displaying TAF2 (green) and TAF10 (red).

With the objective to better understand human TFIID assembly and in particular the incorporation of TAFs into holo–TFIID, we carried out immunoprecipitations from HeLa cell cytoplasmic and nuclear extracts. To test the role of TAF2 in the assembly process, we raised a polyclonal antibody using highly purified recombinant human TAF2 protein for the immunization procedure. We ascertained specificity of the purified antibody against recombinant TAF2 and endogenous TFIID by western blotting (Fig. 1a and Supplementary Fig. 1a). Using this antibody, we carried out co-immunoprecipitation experiments of endogenous TAF2 from the cytoplasm, where TAF2 is synthesized *de novo*, and from nuclear extracts, where TAF2 likely functions in the context of TFIID. To identify proteins that co-precipitated with TAF2 we subjected the immunoprecipitated samples to proteomics analysis by using the multidimensional protein identification technology (MudPIT). MudPIT analysis of proteins co-precipitated with TAF2 from the nuclear fraction revealed the full set of TFIID components (Fig. 1b and Supplementary Table 1). We observed differences in abundance of the individual TAFs, which may argue for the presence of distinct TAF2-associated TAF or TFIID-like complexes in the nucleus. Strikingly, MudPIT analysis of TAF2-associated proteins from the cytoplasmic fraction identified only TAF8 and TAF10, whereas none of the other TAFs could be detected (Fig. 1b,c). We confirmed the presence of TAF2, TAF8 and TAF10 in the cytoplasm of HeLa cells by immunofluorescence experiments (Fig. 1d,e and Supplementary Fig. 1b). These data suggest that a unique endogenous TAF2–TAF8–TAF10-containing TFIID building block exists in the cytoplasm.

### TAF8 nucleates the TAF2–8–10 complex

**Figure 2:**
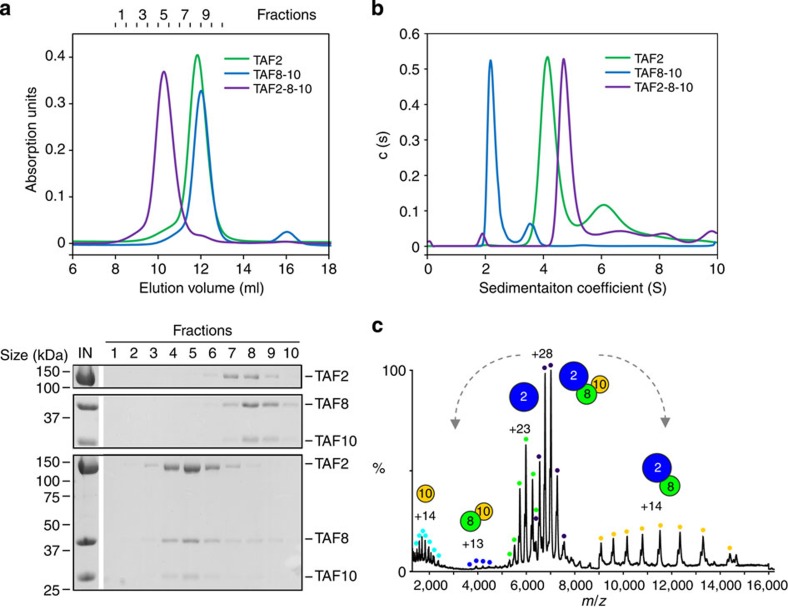
Recombinant TAF2–8–10 complex. (**a**) TAF2, the TAF8–10 pair and a mixture of TAF2–8–10 were analysed by SEC. Elution profiles of TAF2 (green), TAF8–10 (blue) and TAF2–8–10 (purple) are plotted in relative absorption units at 280 nm versus elution volume (top). Fractions are numbered (top of graph). SDS–PAGE analyses of the eluted samples are shown (below). Molecular masses of protein standards are indicated on the left of gel sections. Protein denominations are shown on the right. First lane shows the SEC input (IN). (**b**) Absorbance c(s) profiles from sedimentation velocity analytical ultracentrifugation experiments are plotted for TAF2 (green), TAF8–10 (blue) and TAF2–8–10 (purple). (**c**) Mass spectrum of TAF2–8–10 complex electrosprayed from an aqueous ammonium acetate solution under high collision energy for subunit dissociation. The MS spectrum reveals peaks with corresponding masses for a TAF8–10 dimer (blue dots), TAF2 subunit (green dots) and a predominant TAF2–8–10 complex (purple dots) centred at 4,000, 6,000 and 7,500 *m*/*z*, respectively. At 12,000 *m*/*z* is a TAF2–8 dimer (yellow dots) resulting from the dissociation of the TAF10 subunit (light blue dots) from the intact TAF2–8–10 complex. Proteins and protein complexes are shown schematically as coloured circles.

To further analyse this TAF2–TAF8–TAF10 complex, we used highly purified recombinant human TAF2, TAF8 and TAF10 to reconstitute TAF2–8–10 *in vitro*. We produced recombinant TAF2 and the TAF8–10 pair separately in insect cells and tested complex formation by size-exclusion chromatography (SEC) experiments. SEC of a stoichiometric mixture of TAF2 and TAF8–10 showed a clear peak shift in retention volume towards earlier fractions as compared with the individual components (Fig. 2a). Analysis of the chromatographic fractions by SDS-polyacrylamide gel electrophoresis (SDS–PAGE) shows that all three polypeptides co-elute in the same fractions (Fig. 2a). We observed unusually high molecular weight estimates for the components TAF2 and TAF8–10, and also for the complete TAF2–8–10 complex, which exceed the calculated molecular weights of the proteins. These high estimates can be due to either oligomerization or elongated shapes of the specimens analysed. We therefore determined the oligomeric states of purified TAF2, TAF8–10 and the TAF2–8–10 complex by analytical ultracentrifugation sedimentation velocity and native mass spectrometry (MS) experiments. Sedimentation coefficients of 4.3 S, 2.3 S and 4.9 S were obtained for TAF2, TAF8–10 and TAF2–8–10, respectively (Fig. 2b). Continuous size-distribution analyses returned best-fit molecular weights of 140, 52 and 200 kDa. These values are in good agreement with monomeric TAF2, heterodimeric TAF8–10 and heterotrimeric TAF2–8–10 complexes, with subunit stoichiometries of 1:1 and 1:1:1 in case of the complexes.

Analysis of TAF2–8–10 by native MS revealed a predominant complex with an average molecular mass of 195,797 Da corresponding to a TAF2–8–10 heterotrimer containing one copy of each protein (Supplementary Fig. 2a and Supplementary Table 2). We subjected the TAF2–8–10 complex to collision-induced dissociation (CID) experiments in the mass spectrometer to probe for subunit interactions^37^. The resulting spectra reveal dissociation of the trimeric complex into TAF2–8 and TAF10 submodules (Fig. 2c and Supplementary Table 2). Notably, TAF2 and TAF10 do not interact under the conditions studied, since we did not observe a TAF2-10 species (Fig. 2c). We conclude from these data that TAF2, 8 and 10 assemble as a heterotrimeric complex with a 1:1:1 stoichiometry and that the complex is nucleated by TAF8 and stabilized by distinct TAF2–8 and TAF8–10 interactions.

### TAF8 and TAF10 adopt a non-canonical histone fold dimer

We next dissected the interactions identified by CID. First, we determined the X-ray crystal structure of the TAF8–10 complex. Previous GST pull-down experiments suggested that the interaction between TAF8 and TAF10 is mediated by their HFDs, which are present in the amino-terminal (N-terminal) half of TAF8 and the C-terminal half of TAF10 (refs 28, 30). We co-expressed and purified full-length TAF8–10 complex in insect cells from a polyprotein construct^38^, subjected the complex to limited proteolysis and defined the core complex to TAF8 residues 1–134 and TAF10 residues 98–218 (hereafter referred to as TAF8ΔC and TAF10ΔN, respectively; Supplementary Fig. 3a).

We prepared this TAF8ΔC–TAF10ΔN core complex, but only obtained crystals diffracting X-rays to 5–6 Å resolution. We therefore tested various N- and C-terminal deletion constructs of the two proteins in crystallization experiments. A complex of TAF8–10 comprising TAF8 residues 25–120 and TAF10 residues 112–212 yielded crystals, which diffracted incident X-rays to 1.9 Å resolution (Supplementary Fig. 3b). We determined the structure of this complex by the Sulfur-SAD method and refined the model to a crystallographic R value of 20.5% and a free R factor of 23.7% with excellent stereochemistry (Table 1). The final model includes TAF8 residues 28–120 and TAF10 residues 113–212 with the exception of a flexible loop in TAF10 comprising residues 178–191.

**Figure 3:**
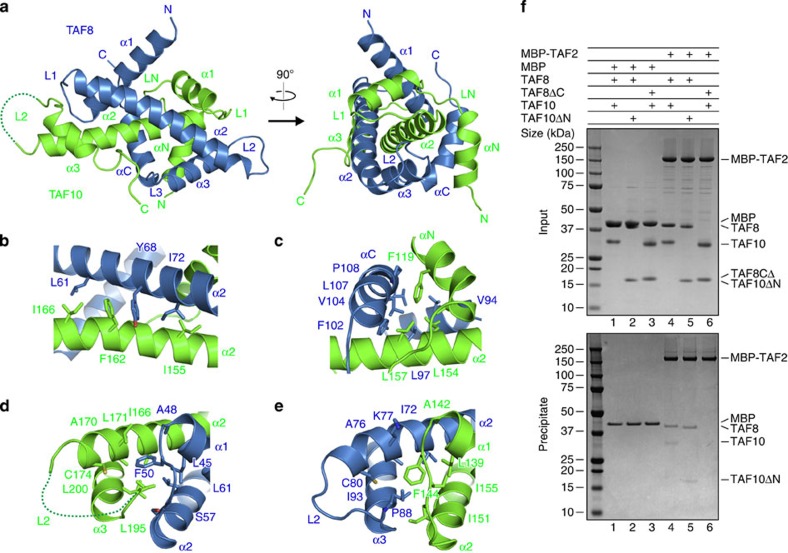
TAF8–TAF10 interactions. (**a**) Crystal structure of human TAF8–10 complex is depicted in a cartoon representation. Two orientations related by a vertical rotation of 90° are shown. TAF8 is coloured in blue and TAF10 in green. The disordered L2 loop of TAF10 is represented by a dotted line. Secondary structure elements and loops are labelled. The TAF8–TAF10 complex adopts a non-canonical HFD pair. (**b**–**e**) Close-up views of the interactions between TAF8 and TAF10. Key interacting residues are highlighted. All structure drawings were generated with PyMOL ( http://www.pymol.org/). (**f**) Pull-down experiments of TAF2 fused to MBP analysing the interactions with TAF8–10, TAF8–TAF10ΔN and TAF8ΔC–TAF10 HFD pairs (see main text for details). Unfused MBP is included as a control. Input samples (top) and samples precipitated on amylose resin (bottom) were resolved on 4–12% gradient gels. Protein identities are shown on the right.

The crystal structure of the TAF8–10 complex reveals that the two proteins adopt atypical HFDs with three central α helices flanked by additional N- and C-terminal α helices (Fig. 3a). In our structure, TAF8 wraps entirely around the α2 helix of TAF10 markedly enveloping its interaction partner (Fig. 3a). Complex formation buries 2212.3 Å^2^ with predominantly hydrophobic intermolecular contacts. As observed in other HFD interactions, the two opposing aromatic residues Y68 of TAF8 and F162 of TAF10 at the crossover of the α2 helices contact each other via hydrophobic stacking interactions and categorize the complex to the H3/H4 family of HFD-containing proteins (Fig. 3b and Supplementary Fig. 3c)^39^. The additional N-terminal α-helix of TAF10, αN and the C-terminal α-helix of TAF8, αC, contact each other on one side of the HFD in a head-to-tail fashion and significantly stabilize the complex by hydrophobic interactions centred on F119 of TAF10 (Fig. 3c).

Interestingly, the proteins TAF8 and TAF10 have similar L1 loop geometries, which are not found in other structures of related HFD-containing TAFs (Fig. 3d,e)^22^,^25^. In both proteins, a phenylalanine of loop L1 (F50 in TAF8 and F144 in TAF10) is embedded in a composite, hydrophobic cavity mainly formed by residues from helices α1/α2 of one protomer and helices α2/α3 of the other (Fig. 3d,e). The amino acids forming this hydrophobic cavity are remarkably similar in TAF8 and TAF10, suggesting an evolutionary interrelation between the two proteins (Supplementary Fig. 3d). To test the functionality of the TAF10 HFD and chimeric mutants thereof, we performed complementation assays in TAF10 null mouse F9 cells^40^. Interestingly, the human TAF10 HFD (residues 116–218) is fully functional in the complementation assay, whereas chimeric constructs, in which either the N-terminal region of TAF10 (residues 116–150) or the C-terminal region (residues 151–218) was substituted by sequences of the yeast TAF10 homologue, were not functional (Supplementary Fig. 3f,g).

Primary sequence comparison with two other TAF10-interacting proteins, TAF3 and human SPT7L, shows that similar residues can be also found in their HFDs^30^, arguing for a conserved binding mode of these proteins known to interact with TAF10 (Supplementary Fig. 3d). Our structure underscores that HFDs in TAFs can adopt a variety of conformations, which may differ significantly from the canonical histone pairs found in the nucleosome.

### HFDs of TAF8 and TAF10 are dispensable for TAF2 binding

We next analysed the physical interactions between TAF2 and the TAF8–10 heterodimer. We first tested the effects of deleting the intrinsically unstructured regions of TAF8 and TAF10 on TAF2 binding in pull-down assays with purified proteins. As a control, full-length TAF8–10 was co-precipitated with TAF2 tagged with maltose-binding protein (MBP; Fig. 3f). Truncation of the N-terminal region of TAF10 (TAF8–TAF10ΔN, TAF10 residues 98–218) did not change the binding properties and still co-precipitated with MBP–TAF2. In contrast, a truncated complex of TAF8–10, in which the flexible C-terminal region of TAF8 was deleted (TAF8ΔC–TAF10, TAF8 residues 1–134), did not co-precipitate with MBP–TAF2 suggesting that the region that mediates binding to TAF2 resides in the C-terminal, low-complexity tail of TAF8 (Fig. 3f). We confirmed the interaction between TAF2 and the C-terminal tail of TAF8 by SEC. We utilized full-length TAF2 and a fusion protein of MBP with residues 105–310 of TAF8 and evidenced complex formation (Supplementary Fig. 4). These results are consistent with the CID data in native MS, which showed that only TAF8, and not TAF10, is directly interacting with TAF2.

### TAF2 recognizes short motives in the TAF8 C-terminal region

**Figure 4:**
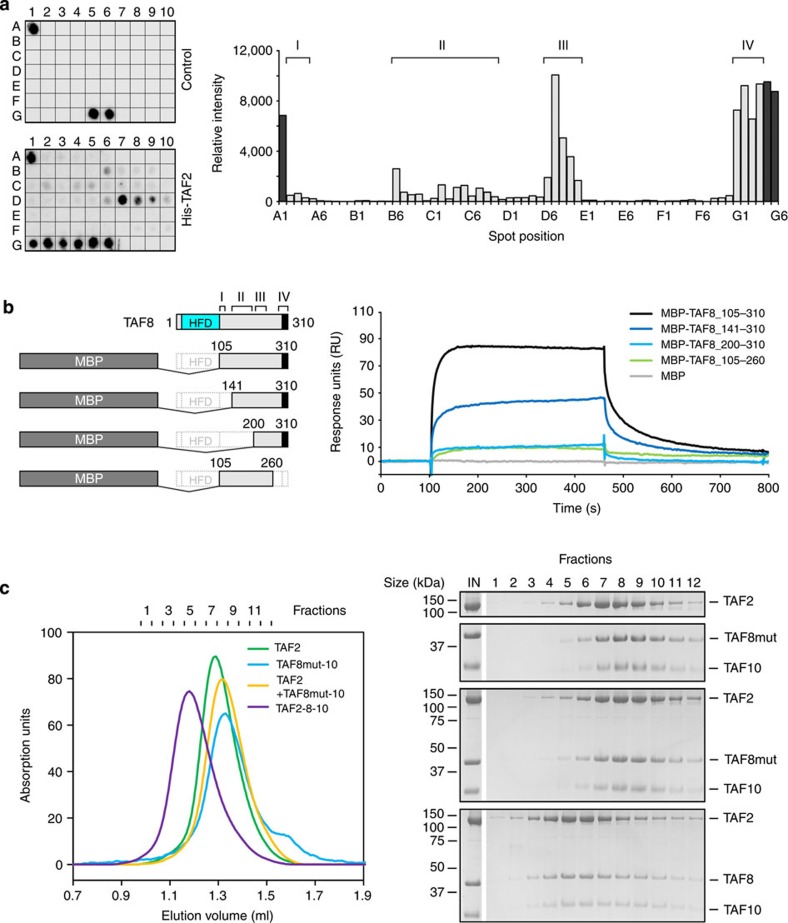
TAF8–TAF2 interactions. (**a**) His-tagged TAF2 binding to overlapping peptides of the TAF8 C-terminal region (residues 105–310) spotted onto nitrocellulose membranes (spots A2-G4, left) was analysed by utilizing a peptide array. Bound TAF2 was visualized by luminol reaction and signal intensities were plotted for each spot after background subtraction (right). Spots A1, G5 and G6 served as positive controls. TAF2 protein was omitted for the control membrane. The four major binding regions (I–IV) are indicated above the histogram. (**b**) SPR experiments with immobilized full-length TAF2 as ligand and MBP (control) as well as MBP fusions of TAF8 fragments 105–310, 141–310, 200–310 and 105–260 as analytes. TAF8 deletion constructs are schematically shown as bar diagrams (left). TAF2-interacting regions on TAF8 as identified in **a** are highlighted. SPR sensorgrams at identical analyte concentrations of 500 nM are plotted as RU versus time (right). (**c**) SEC analyses assessing the influence of TAF8 point mutations on TAF2 binding. Elution profiles for the indicated proteins and protein complexes are plotted on the left and SDS–PAGE analyses of each run are shown on the right. Molecular masses of protein standards are denoted on the left of the gels and protein names on the right.

We characterized the TAF2–TAF8 interaction further by means of a peptide array. We monitored the binding of His-tagged TAF2 to peptide arrays covering residues 105–310 of TAF8 (Fig. 4a). Densitometric analysis of the arrays indicated that TAF2-binding clusters around four distinct regions; a short N-terminal region I covering TAF8 residues 105–125, a less well-defined region II including residues 147–202 and regions III and IV spanning residues 207–238 and 282–310, respectively (Fig. 4a).

We next analysed the individual contributions of these four TAF8 regions to TAF2 binding by surface plasmon resonance (SPR) experiments. We generated N- and C-terminal deletion constructs of TAF8 and fused them to MBP (Fig. 4b). We monitored the association and dissociation phases of the MBP–TAF8 truncations on TAF2-charged sensor chips and compared binding kinetics at identical analyte concentrations. An MBP–TAF8-fusion construct spanning the entire C-terminal region (TAF8 residues 105–310) showed a maximal association level of about 85 response units (RU) with fast on and off rates (Fig. 4b). A shorter MBP-fusion protein lacking region I (TAF8 residues 141–310) showed similar kinetics but a reduced maximal association level of ~40 RU (Fig. 4b). MBP-fusion constructs with deleted regions I and II or IV (TAF8 residues 200–310 or 105–260, respectively) hardly interacted with immobilized TAF2 showing maximal association levels of less than 10 RUs (Fig. 4b). These data indicate that all four TAF2-interacting regions of TAF8 contribute cooperatively to the binding to TAF2.

On the basis of our peptide array and SPR results, we introduced TAF8 point mutants into the TAF8–10 polyprotein expression construct by substituting three triple amino-acid clusters spanning residues 185–187 (DVE), 222–224 (PYL) and 293–295 (PYL) with alanines. We produced and purified wild-type TAF8–10 and the mutated TAF8–10 complex and analysed TAF2 binding via SEC (Fig. 4c). In contrast to wild-type TAF8–10, formation of a trimeric TAF2–8–10 complex was not observed with the three triple amino-acid cluster TAF8–10 mutant, corroborating the results that we obtained with our peptide array and SPR experiments (Fig. 4c).

### TAF8 promotes TAF2 incorporation in TFIID

**Figure 5:**
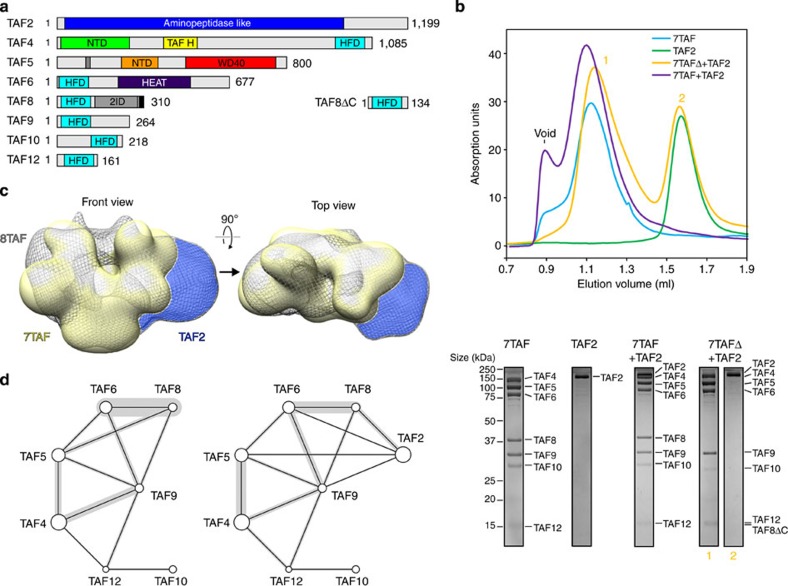
TAF8 promotes TAF2 incorporation in TFIID. (**a**) TFIID components studied are shown as bar diagrams. Predicted low-complexity regions of the proteins are coloured in grey. A black bar denotes the TAF8 NLS. C-terminally truncated TAF8 (TAF8ΔC), which was used to reconstitute the 7TAFΔ complex, is depicted on the right. Numbers denote first and last amino acids for each protein. (**b**) Impact of the TAF8 truncation on TAF2 binding to 7TAF complexes. SEC elution profiles for indicated proteins and protein complexes are shown (top). Corresponding SDS–PAGE gel sections of peak fractions of each run are shown (bottom). Protein size markers are shown on the left; protein identities on the right. (**c**) Three-dimensional single-particle EM reconstruction of negatively stained 8TAF complex (grey mesh) superimposed on 7TAF complex (yellow, from ref. 12) is shown in two views related by a 90° rotation as indicated (arrow). Difference density attributed to bound TAF2 is highlighted in blue. (**e**) Protein–protein crosslink maps for the 7TAF complex (left) and the 8TAF complex (right) are shown. Circle sizes represent relative molecular weights of each protein. Black lines connect crosslinked proteins. Grey bars superimposed on black lines indicate crosslink frequencies ( www.crosslinkviewer.org). Original images corresponding to the gel sections shown in Fig. 5b (bottom panel) are provided in Supplementary Figure 8.

We showed recently that a TFIID subcomplex comprising TAF4, 5, 6, 8, 9, 10 and 12 (hereafter referred as 7TAF) can be formed *in vitro* by binding TAF8–10 to a physiological nuclear core–TFIID complex, which constitutes an important intermediate in holo–TFIID assembly^12^,^21^. We next asked whether the association of TAF2 to this 7TAF complex depends on the C-terminal region of TAF8, which we identified as responsible for TAF2 binding in the TAF2–8–10 complex. We produced and purified recombinant 7TAF and a 7TAFΔ complex, in which TAF8 is substituted by TAF8ΔC (Fig. 5a). We monitored binding of a mCherry-TAF2 fusion protein to 7TAF and 7TAFΔ complexes using SEC. We introduced the mCherry tag on TAF2 to unambiguously separate the protein from TAF4 in SDS–PAGE. In all SEC experiments, we used stoichiometric amounts of TAF2 in relation to TAF8–10 or the truncated TAF8ΔC–TAF10 complex. Interestingly, TAF2 could be fully incorporated into the 7TAF complex, whereas TAF2 did not interact noticeably with the 7TAFΔ complex, in which the C-terminal TAF2-interaction region of TAF8 had been deleted (Fig. 5b).

Next we mapped the position of TAF2 on 7TAF. To this end, we determined a three-dimensional model of negatively stained 7TAF complexes bound to TAF2 (hereafter referred as 8TAF complex) by single-particle electron microscopy and compared the resulting structure to the reconstruction of the 7TAF complex we had determined previously^12^. We observed major density differences clearly positioned on only one side of the particle, indicating TAF2 location (Fig. 5c and Supplementary Fig. 5a). Interestingly, our 8TAF complex reconstruction resembles a precursor to the characteristic clamp shape of holo–TFIID, in contrast to the less elongated shape of 7TAF and core–TFIID^10^ (Supplementary Fig. 5b).

Next we sought to characterize possible alterations in the protein–protein interaction networks along the assembly pathway to holo–TFIID. In particular, we looked at the transition from 7TAF to 8TAF complexes on TAF2 binding by crosslinking and MS (CLMS) experiments. We crosslinked 7TAF and 8TAF complexes with the bifunctional reagent bis(sulfosuccinimidyl)suberate, BS3 that targets mostly lysines^41^ (Supplementary Fig. 6a). Crosslinked complexes were separated from non-crosslinked species by SDS–PAGE, in-gel digested and crosslinked peptides were assigned to ion masses observed by MS. We identified 37 protein–protein crosslinks for the 7TAF complex and 37 protein–protein crosslinks for the 8TAF complex with an overlap of 21 crosslinked peptides between the two complexes (Fig. 5d, Supplementary Fig. 6b–e and Supplementary Table 3). Our data suggest that TAF9 plays a central role in 7TAF complex architecture by interconnecting TAF4, 5, 6, 8 and 12 (Fig. 5d). In our CLMS data, prominent crosslinks between TAF8 and TAF10 were not present, consistent with the paucity of lysines within crosslinking distance, and the partly buried location of the TAF8–10 dimer within the 7TAF complex^12^. In the 8TAF complex, we observed crosslinks of the C-terminal region of TAF8 with residues on TAF2, which are predicted to map to the surface (Fig. 5d). In addition to its proximity to TAF8, TAF2 is also positioned closely to TAF5, 6 and 9 and promotes crosslinking between TAF4 and 5 (Fig. 5d). Our data indicate that TAF2 is indeed anchored to the 7TAF complex via the TAF2-interacting region on TAF8 and that binding of TAF2 induces significant conformational changes that result in novel TAF–TAF interactions not present in the 7TAF complex.

### TAF2–8–10 binds Importin α1 via the TAF8 NLS

**Figure 6:**
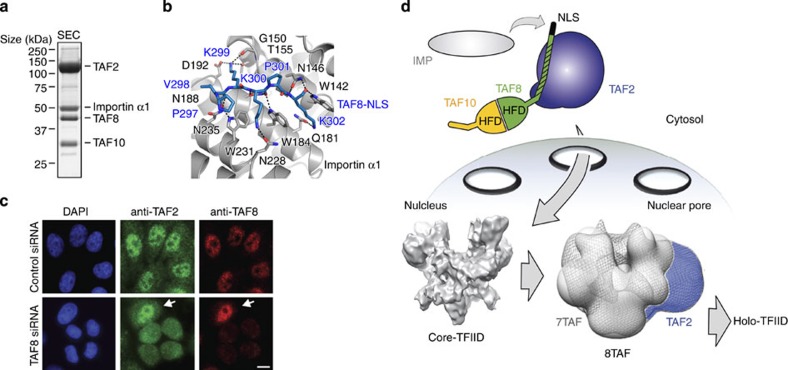
Nuclear TFIID assembly from preformed submodules. (**a**) A complex consisting of TAF2, TAF8, TAF10 and Importin α1^ΔIBB^ was formed from highly purified components. Importin α1^ΔIBB^ was mixed in a twofold molar excess with purified TAF2–8–10 and the mixture purified by SEC. SDS–PAGE analysis of the peak fraction is shown. (**b**) Importin α1-TAF8 complex crystal structure. Magnified view of interacting residues of the major binding site of Importin α1 (grey) with residues of the NLS of TAF8 (blue). Importin α1 is shown in ribbon representation and the TAF8–NLS as a stick model. TAF8 residues R303 and R304 are not involved in contacting Importin α1 and are omitted for clarity. (**c**) Immunofluorescence microscopy of HeLa cells depleted of TAF8 (TAF8 siRNA) by RNAi or control cells (Control siRNA). Nuclei are visualized by 4′,6-diamidino-2-phenylindole (DAPI) staining (blue). TAF2 is displayed in green and TAF8 in red. Arrows point to a non-transfected cell. Scale bar, 10 μm. (**d**) Cartoon model of cytoplasmic TAF2–8–10 complex and nuclear holo–TFIID assembly. The NLS of TAF8 is filled in black. The TAF2-interaction domain within TAF8 is highlighted by shading. TAF2, blue; TAF8, green; TAF10, orange. The TAF2–8–10 complex resides in the cytoplasm, whereas the physiological symmetric core–TFIID complex is found in the nucleus^10^,^22^. The cryo-electron microscopy density envelope of core–TFIID complex is shown (adapted from ref. 12). On binding of Importin α1 (grey) to the TAF8 NLS, TAF2–8–10 translocates into the nucleus through a nuclear pore (arrow). In the nucleus, Importin α1 is released and TAF2, 8 and 10 associate with core–TFIID, to form intermediates including the asymmetric 8TAF complex along the holo–TFIID assembly pathway.

Biochemical and cell biology experiments demonstrated that the C-terminal NLS within TAF8 is necessary for shuttling TAF8 and TAF10 from the cytoplasm to the nucleus in an Importin α/β-dependent fashion^30^. We asked whether the identified endogenous TAF2–8–10 complex would be capable of recruiting Importin α1 *in vitro* to form a nuclear import complex. To this end, we mixed highly purified TAF2–8–10 with a twofold molar excess of an Importin α1 variant lacking the Importin β-binding domain (Importin α1^ΔIBB^). We observed efficient complex formation in SEC indicating that Importin α1^ΔIBB^ was stoichiometrically incorporated into the TAF2–8–10 complex (Fig. 6a and Supplementary Fig. 7a). We also observed by SEC that TAF2 alone is not bound by Importin α1.

To define the binding region between Importin α1 to the TAF2–8–10 complex, we determined the X-ray crystal structure of the C-terminal NLS of TAF8 in complex with Importin α1^ΔIBB^ at 1.75 Å resolution. The refined model has a crystallographic R value of 15.3% and a free R factor of 18.0% with good stereochemistry (Table 1). Importin α1 residues 72–497 and residues 297–305 of the TAF8 peptide could be unambiguously traced in the electron density map. The TAF8 peptide binds as a monopartite NLS via residues 297–302 (Fig. 6b). In previous Importin/NLS structures, asparagines N146, N188 and N235 of Importin α1 hydrogen bond to NLS main chain amide and carbonyl groups at positions P1, P3 and P5 (ref. 42) (Fig. 6b and Supplementary Fig. 7b). Importin α1 tryptophanes W142, W184 and W231 form apolar pockets, which accommodate the aliphatic moieties of lysine residues K300 and K302, and position the TAF8 NLS backbone via residues P297, K300 and K302 (Fig. 6b). The side chain of K299 of TAF8 is coordinated by the main chain carbonyl group of G150, the hydroxyl group of T155 and the carboxylate of D192, whereas side chains of K300 and K302 of TAF8 are contacted by side chain carbonyl groups of N228 and Q181, respectively. We could also model a less well-defined short amino-acid segment at the minor binding site of Importin α1 (Supplementary Fig. 7c). To assess whether the minor binding site of Importin α1 plays a role in binding the NLS of TAF8, we determined the kinetic parameters for the Importin α1/TAF8–NLS complex formation by isothermal titration calorimetry. Using Importin α1^ΔIBB^ as an analyte and an NLS peptide comprising TAF8 residues 288 to 310 as titrant, we obtained a 1:1 binding stoichiometry with a dissociation constant in the low micromolar range (*K*_d_=10.4+/−0.8 μM; Supplementary Fig. 7d). In accordance with our crystal structure, the binding of the NLS of TAF8 to Importin α1 is driven by enthalpy involving mainly hydrogen bonds and van der Waals interactions (enthalpy change of ΔH=−18.5+/−1.3 kcal mol^−1^ and entropy change of −TΔS=11.5 kcal mol^−1^).

We next asked if the nuclear localization of TAF2 is dependent on the presence of TAF8 *in vivo*. Therefore, we knocked down endogenous TAF8 in HeLa cells by RNA interference (RNAi) treatment for 48h and compared the nuclear/cytoplasmic distribution of TAF2 in TAF8 knockdown cells with control cells by immunofluorescence (Fig. 6c). Short interfering RNA (siRNA) treatment leads to depletion of TAF2 in the nucleus and to an enrichment of TAF2 in the cytoplasm, suggesting that the import of TAF2 is controlled by TAF8 (Fig. 6c).

Taken together, our data suggest the presence of a nuclear import particle in which the TAF2–8–10 complex is bound by the major binding site of Importin α1 via the NLS of TAF8, poised to shuttle into the nucleus (Fig. 6d). On release of Importin α1, the TAF2–8–10 module then combines with core–TFIID (Supplementary Fig. 2b,c). Association of these preformed TFIID submodules leads to conformational rearrangements in the resulting intermediate TAF complex which enables formation of the functional nuclear holo–TFIID (Fig. 6d).

## Discussion

Elucidation of the structure and function of multiprotein complexes in gene regulation is an intense focus of current research efforts^43^,^44^. Whereas three-dimensional models of fully assembled multiprotein complexes derived from X-ray crystallography or single-particle cryo-electron microscopy provide a wealth of information on the architecture of such complexes, little is known about how the cell controls and regulates the ordered assembly of multiprotein gene regulatory complexes such as TFIID.

Several earlier studies described the existence of a variety of TFIID complexes with distinct subunit composition in different cell types^23^,^45^,^46^,^47^,^48^ conveying a concept of modular TFIID assembly. To gain more insights into the regulated assembly of TFIID, and to try to understand how the regulated assembly of such complexes may contribute to gene regulation, we initiated a series of experiments to identify TFIID assembly intermediates in the cytoplasm, and different TFIID assemblies in the nuclei of human cells. In the framework of these experiments, we were also aiming to uncover the incorporation pathway of TAF2 in TFIID. We identified a novel TFIID building block comprising TAF2–TAF8–TAF10 in the cytoplasm of human cells. We also characterized the interactions stabilizing this cytoplasmic complex in an integrated approach combining native MS, X-ray crystallography, SPR, peptide arrays and biochemical and biophysical methods. Our experiments indicate that TAF2 interacts with the C-terminal unstructured region of TAF8 *in vitro*, substantiating protein–protein interaction mapping experiments of *Saccharomyces cerevisiae* TFIID^49^.

Previously, it was shown that TAF8 shuttles from the cytoplasm to the nucleus in an Importin α/β-dependent pathway and piggybacks TAF10 into the nucleus^30^. Owing to the lack of a NLS, TAF10 cannot translocate to the nucleus on its own and depends on the NLS of its interaction partner, TAF8 (ref. 30). Deletion mutants suggested that Importin binding resides in the extreme C-terminus of TAF8 (ref. 30). We show that a tetrameric complex consisting of Importin α1, TAF2, TAF8 and TAF10 can be assembled from purified components *in vitro* suggesting a co-import mechanism for the three proteins. Our TAF2 and TAF10 cellular localization experiments support this mechanism, indicating that knockdown of TAF8 by RNAi not only alters the cellular localization of TAF10, but likewise the localization of TAF2.

To define the precise modes of interaction between Importin α1, TAF8 and TAF10, we solved the X-ray crystal structures of Importin α1 bound to the NLS of TAF8 on one hand, and of the HFD pair formed by TAF8 and TAF10 on the other. The crystal structure of the TAF8–10 complex reveals atypical histone folds of the two proteins and shows a combination of symmetric and asymmetric structural elements. Both TAFs share characteristic conformations of their L1 loops, which give rise to pseudosymmetric structures at the extremities of their HFDs. Otherwise, the presence of additional αN and αC helices render the TAF8–10 complex asymmetric. The pseudosymmetric L1 loops are characteristic for the TAF8–10 complex, since similar arrangements cannot be found in the crystal structures of the *Drosophila* TAF6–TAF9 and the human TAF4–TAF12 complex^22^,^25^ suggesting that the overall shape and precise geometry of the complex is important for integration into core–TFIID.

The crystal structure of Importin α1 with the NLS of TAF8 reveals that TAF8 has a classical short monopartite NLS, which is recognized by the major binding site of Importin α1. Interestingly, phosphorylation of conserved serine residues C-terminal to canonical NLSs of different nuclear proteins either enhance, or abolish, the binding affinity of different importins, thus regulating nuclear import^50^,^51^,^52^. Similarly, phosphorylation may also fine-tune nucleocytoplasmic shuttling of the TAF2–8–10 complex. Two serine residues predicted to be phosphorylated are located C-terminal to the NLS of TAF8. Therefore, the affinity of TAF8 to Importin α1 and consequently the nuclear import could be modified by phosphorylation. It will be interesting to see if such a regulatory mechanism by post-translational modification exists for the import of the TAF2–8–10 complex *in vivo*. Our crystal structure of Importin α1 bound to the TAF8 NLS further suggests that the cytosolic TAF2–8–10 complex together with Importin α1 constitutes an import particle responsible for delivering this building block into the nucleus. Likewise, our experiments indicate that this TAF2–8–10 building block is responsible for the incorporation of TAF2 in nuclear TFIID.

A stable TFIID core complex comprising two copies each of TAF4, 5, 6, 9 and 12 was identified in *Drosophila* and human cell nuclei^12^,^21^. Previously, we postulated that the binding of TAF8–10 causes a rearrangement of the symmetric TFIID core complex to an asymmetric particle, which is then capable of accommodating the remaining TAFs and TBP, each in single copy^12^. We propose that association of the TAF2–8–10 complex with the preassembled nuclear core–TFIID involves an intricate network of interactions between the TAF8 C-terminal tail and TAF2 on one hand, and the globular HFD pair of the TAF8–10 complex and core–TFIID on the other. Our current data suggest that TAF8–10 may function alike a chaperone to regulate nuclear import and integration of TAF2 into core–TFIID. Note, however, that in the cytoplasmic extracts, apart from TAF8 and TAF10, we did not detect any of the other TFIID components stably associated with endogenous TAF2. Therefore, we hypothesize that TAF1, 7, 11, 13 and TBP incorporate into the TFIID structure probably at a defined, later step, after TAF2–8–10 has been accreted. Moreover, notwithstanding the fact that we did not find either TAF1 or TBP associated with TAF2 in the cytoplasm in our co-IP coupled MS analyses, it still can be envisioned that the TAF2–8–10 complex is capable of nucleating the formation of the holo–TFIID complex, including the TAF1–TBP module, and thus promote transcription from Inr-containing core promoters.

Interestingly, TAF2-containing and TAF2-lacking, as well as TAF10-containing and TAF10-lacking, TFIID complexes have also been extracted from human cells^32^,^34^,^45^. Thus, in good agreement with the modular TFIID assembly concept, our observations suggest that the here characterized TAF2–8–10 building block would not always incorporate in all canonical TFIID complexes but, even in the nucleus, may exist as an independent regulatory entity. Future experiments will be required to elucidate the function(s) of holo–TFIID complexes versus complexes lacking TAF2–8–10. Along the same lines, it will also be interesting to test whether a TAF2–8–10 complex alone or in combination with core–TFIID can modulate transcription efficiency of Inr-dependent genes. Promoter architecture may at this junction control transcription regulation by gauging the assembly rate of holo–TFIID from building blocks^48^. From a pharmaceutical point of view, it is to date entirely unclear whether or not neurological disorders caused by mutations in the *TAF2* gene develop due to altered regulation of transcriptional activity or due to other currently unknown mechanisms. Future experiments will be required to elucidate if these TAF2 mutations may actually affect TAF8 binding and TFIID assembly.

Our results support the view that stable partial TFIID complexes—that potentially have important functions of their own—might exist in the cell. These complexes may represent functional cytoplasmic or nuclear modules, which assemble into holo–TFIID in a stepwise fashion. Also, our results point to an important role of cytoplasmic–nuclear transport in holo–TFIID formation. We anticipate that such processes will likewise play important roles in regulating the assembly and activities of many other multiprotein complexes that direct gene transcription.

## Methods

### DNA constructs

Cloning of TAF2, TAF8 and TAF10 expression constructs in MultiBac plasmids pPBac^38^, pFL and pIDC^53^ is detailed in the Supplementary Methods. Expression plasmids for subcomplexes TAF5–6–9 (pPBac-3TAF) and TAF4–12 (pDiFB-412) and Importin α1^ΔIBB^ were described previously^12^,^54^. Truncated Importin α1 (residues 71–497) was generated by amplifying the coding region of Importin α1^ΔIBB^ by PCR. All constructs were verified by DNA sequencing.

### Sequence alignments

Alignments were generated using the ClustalW2 server^55^ and plotted with ESPript ( http://espript.ibcp.fr)^56^. Protein sequences for human TAF3 (UniProt accession number Q5VWG9) and human SPT7L (O94864) were retrieved from the UniProt server ( www.uniprot.org).

### Protein production and purification

MBP–TAF8-fusion proteins were produced in *E. coli* Rosetta (DE3) cells (Novagen) and purified by metal affinity chromatography using TALON resin (Clontech) followed by size-exclusion chromatography on a Superdex 200 16/60 column (GE Healthcare; detailed in Supplementary Methods). Importin α1 constructs (residues 60–529 or residues 71–497) were produced and purified as described^54^, except that *E. coli* Rosetta (DE3) cells (Novagen) were used. Production and purification of core–TFIID and 7TAF complexes was performed as described^12^.

Proteins TAF2, MBP–TAF2, mCherry-TAF2 and TAF8–10 complex were produced using the MultiBac system^53^. Expressed protein was captured via TALON resin (Clontech) from the cell lysate in batch. Proteins were further purified by ion exchange chromatography using a 5-ml SP-Sepharose HiTrap column (GE Healthcare) followed by gel filtration using Superdex200 10/300 or Superose6 10/300 columns (GE Healthcare; Supplementary Methods). Proteins were flash frozen in liquid nitrogen and stored at −80 °C in aliquots.

### Binding experiments

SEC experiments were carried out with ÄKTA purifier or ÄKTA Micro systems (GE Healthcare) using Superdex200 10/300, Superdex200 PC3.2, Superose6 PC3.2 or Superose6 PC3.2 Increase columns. Binding experiments shown in Fig. 2a were performed in running buffer comprising 25 mM HEPES-NaOH, pH 7.5, 150 mM NaCl, 1 mM DTT (dithiothreitol). Runs in Figs 4c and 5b and Supplementary Fig. 2b,c were performed in buffer comprising 25 mM HEPES-NaOH, pH 7.5, 500 mM NaCl, 1 mM DTT.

### Crystallization and structure determination

Screening for crystallization conditions was performed at the High Throughput Crystallization (HTX) laboratory (EMBL Grenoble, France; Supplementary Methods). Crystals of truncated TAF8–10 complex (TAF8 residues 25–120 and TAF10 residues 112–212) were refined manually by mixing equal volumes of protein solution containing 15–25 mg ml^−1^ TAF8–10 in 25 mM Tris-HCl, 150 mM NaCl at pH 7.5 and crystallization solution containing 1.4 M Na/K PO_4_ at pH 7.6. Crystals grew in space group P3_1_21 with cell dimensions of *a*=*b*=51.3 Å and *c*=144.8 Å. Crystals were cryoprotected by adding 20% (v/v) glycerol and flash frozen in liquid nitrogen. Diffraction data were collected at 100 K on beamline PROXIMA 1 using a Pilatus 6 M detector (SOLEIL synchrotron, Gif-sur-Yvette, France) and were integrated and scaled using X-ray Detector Software (XDS)^57^. The structure of TAF8–10 was solved by the Sulfur-SAD method. A partial model could be built into the experimental electron density map by iterative rounds of density modification and automated structure building using programs Pirate and Buccaneer from the CCP4i suite^58^. The model was used to phase a high-resolution data set by molecular replacement using Phaser^58^,^59^. Diffraction data were corrected for anisotropy using the Diffraction Anisotropy Server (services.mbi.ucla.edu/anisoscale)^60^ and an isotropic B of −11.99 Å^2^. The TAF8–10 structure was built and refined using programs Coot^61^ and Phenix^62^, respectively, including TLS parameter and individual B-factor refinement.

Crystals of Importin α1 (residues 60–529) with a synthetic TAF8 NLS peptide (amino acids 297-PVKKPKIRRKKSLS-310 (Peptide Specialty Laboratory, Germany) were grown by mixing 2 μl of protein solution containing 8 mg ml^−1^ Importin α1/TAF8–NLS in 10 mM Tris-HCl, pH 8.0, 150 mM NaCl and 1 mM DTT with 1 μl reservoir solution containing 100 mM HEPES-NaOH, pH 7.1, 12% (w/v) polyethylene glycole 3,350 and 200 mM L-proline in sitting drop vapour diffusion plates. Crystals grew in space group P2_1_2_1_2_1_ with cell dimensions of *a*=54.3 Å, *b*=77.7 Å and *c*=128.6 Å. Crystals were cryoprotected by the addition of 30% (v/v) ethylene glycole and flash frozen in liquid nitrogen. Data sets were collected at 100 K on beamline ID14-1 using an ADSC Quantum Q210 detector (European Synchrotron Radiation Facility ESRF, Grenoble, France). Diffraction data were integrated and scaled using XDS^57^. The structure of Importin α1/TAF8–NLS was solved by molecular replacement using Importin α1 (PDB ID 3RZ9) as a search model. The Importin α1/TAF8–NLS structure was built and refined with Coot^61^ and Phenix^62^, respectively, including TLS parameter, occupancy and individual B-factor refinements.

### Surface plasmon resonance

Biosensor experiments were performed at 25 °C on a BIACORE 3000 (Biacore AB, Uppsala). TAF2 ligand was immobilized onto CM5 sensor chips (GE Healthcare) to a level of 2,500 RU using amine-coupling chemistry. Truncation mutants of TAF8 fused C-terminally to MBP were serially diluted into running buffer (25 mM HEPES-NaOH, pH 7.0, 300 mM NaCl, 0.01% (v/v) NP-40). For association phase, 150 μl of analyte at a concentration of 500 nM were injected at a flow rate of 25 μl min^−1^ and dissociation phases were monitored for 200 s by injecting running buffer only. Binding responses were recorded and responses from referencing sensorgrams were subtracted using BIAevaluation software (GE Healthcare). Data were globally analysed with the analysis software.

### Isothermal titration calorimetry

Calorimetric experiments were conducted in duplicates with a MicroCal iTC_200_ instrument (GE Healthcare) at 25 °C. Importin α1 (residues 71–479) and the TAF8 NLS peptide (residues 288-NPYLRPVKKPKIRRKKSLS-310) were extensively dialysed against ITC buffer (25 mM Tris-HCl pH 8.0, 150 mM NaCl, 1 mM β-mercaptoethanol) and used at concentrations of 39 μM and 1.5 mM, respectively. Protein concentrations were determined by absorbance spectroscopy at 280 nm with calculated extinction coefficients of 48,930 M^−1^ cm^−1^ for Importin α1 and 1,490 M^−1^ cm^−1^ for the peptide. TAF8 peptide (1.5 μl) was injected for 3 s with a spacing of 180 s between injections into 200 μl of Importin α1. Heat changes were recorded over 26 injections. Calorimetric titration data were integrated, corrected for heat of dilution of the TAF8 peptide alone and analysed using Origin software version 7.0 according to a one-site binding model. Binding stoichiometry (*n*), association constant (*K*_a_), binding enthalpy (Δ*H*) and entropy change (Δ*S*) were deduced from fitted isotherms by nonlinear regression. Gibbs free energy difference was calculated using the equation Δ*G*=Δ*H*–*T*Δ*S*.

### Pull-down assays

MBP pull-down assays were performed by mixing 10 μg bait (MBP or MBP–TAF2) with 10 μg prey (TAF8–10, TAF8ΔC–TAF10, TAF8–TAF10ΔN) for 1 h at 4 °C in binding buffer (25 mM HEPES-NaOH, pH 7.5, 500 mM NaCl, 5% (v/v) glycerol, 2 mM β-mercaptoethanol). Protein mixtures were incubated with 20 μl Amylose resin (New England Biolabs) for 1.5 h at 4 °C. Resin was washed three times with binding buffer, once with washing buffer (25 mM HEPES-NaOH, pH 7.5, 500 mM NaCl, 5% (v/v) glycerol, 2 mM β-mercaptoethanol, 0.05% NP-40) and again three times with binding buffer. Proteins were eluted in 15 μl binding buffer containing 30 mM D-maltose and analysed by 4–12% Bis-Tris NuPAGE (Invitrogen).

### Limited proteolysis experiments

The TAF8–10 complex (1 mg ml^−1^) was treated with chymotrypsin at an enzyme-to-protein ratio of 1:10 (w/w). Samples were taken after 2, 5, 10, 20, 40 and 60 min and analysed by SDS–PAGE. To identify the TAF8–10 core complex, the proteolysed sample was loaded on a Superdex75 10/300 column (GE Healthcare) before N-terminal sequencing and MS analysis of comigrating polypeptides.

### Peptide arrays

Pepscan libraries of the C-terminal region of TAF8 (residues 105–310) were immobilized on cellulose membranes via double β-alanine anchors and assembled using the SPOT technology (AG Molekulare Bibliotheken, Charité—Universitätsmedizin Berlin, Germany). Overlapping 20-mer peptides of TAF8 were synthesized by Fmoc (9-fluorenylmethoxycarbonyl) chemistry with an offset of three amino acids between neighbouring spots. Low-density hexa-Histidine peptides were used as controls. Pepscan membranes were blocked in blocking buffer (50 mM Tris-HCl, pH 7.6, 500 mM NaCl, 20% (w/v) sucrose, 3% (w/v) bovine serum albumin) for 1 h at 4 °C, washed with TBS (50 mM Tris-HCl, pH 7.6, 500 mM NaCl) and incubated for 1.5 h with His-tagged TAF2 (10 μg ml^−1^) in blocking buffer or with blocking buffer alone. Membranes were incubated with mouse anti-His monoclonal primary antibody (Sigma-Aldrich, catalogue number H1029, dilution 1:3,000) and peroxidase-conjugated anti-mouse secondary antibody (Sigma-Aldrich, catalogue number A5906, dilution 1:10,000) in blocking buffer. Membranes were washed three times with TBS between each incubation step. Luminol solution (Pierce) was added and luminescence detected on a KODAK 4000MM photoimager. Images were analysed using the Dot Blot Analyzer tool in ImageJ.

### Analytical ultracentrifugation

Sedimentation velocity experiments were performed in a Beckman XL-I analytical ultracentrifuge (Beckman Coulter). The purified proteins and protein complexes TAF2, TAF8–10 and TAF2–8–10 were loaded into sapphire-windowed cells with 12-mm optical path length and spun in an An-60Ti rotor (Beckman Coulter). Absorbance at 280 nm was measured for 16 h at 42,000 r.p.m. and 10 °C. The data were analysed in terms of continuous size-distribution (c(s)) with the Sedfit program^63^, considering 200 particles with sedimentation coefficients, *s*, between 0.1 and 20 S. A partial specific volume of 0.73 and frictional ratios of 1.4 (TAF2, TAF2–8–10) and 1.6 (TAF8–10) were used. A regularization procedure with confidence level of 0.68 was applied. Sample densities and viscosities were determined with Sednterp^64^ to 1.023 g ml^−1^ and 1.40 mPa.s (TAF2, TAF2–8–10) and 1.021 g ml^−1^ and 1.31 mPa s (TAF8–10).

### Native MS

Purified TAF2–8–10 complex (30 μl) was buffer exchanged into 500 mM ammonium acetate buffer (pH 7.5) using Amicon spin concentrators (Millipore, 10 kDa MWCO). All MS experiments were performed on a Quadrupole Time-of-flight (Q-ToF) II mass spectrometer (Waters, Manchester, UK) in the positive ion mode^65^. For data acquisition, 2 μl of the sample was injected into the mass spectrometer with gold-coated capillary needles made in-house using a needle puller (Harvard Apparatus, Holliston, MA, USA). MS spectra were acquired using a capillary voltage of 1.7 kV and cone and collision voltages of 100 V. Time-of-flight and analyser pressures were at 5.6 × 10^−6^ and 4.2 × 10^−4^ mbar, respectively. Data sets were acquired and processed with MassLynx V4.1 software (Waters, UK) with minimal smoothing and no background subtraction. The recorded mass spectra were calibrated externally using 100 mg ml^−1^ caesium iodide in water.

### CLMS analyses

7TAF complexes were produced as described^12^. 8TAF complexes were reconstituted from purified 7TAF complexes by adding twofold molar excess of TAF2 and removal of unbound TAF2 by SEC. 7TAF (200 μg) and 8TAF complexes (200 μg) were crosslinked by BS3 (Bis-sulfosuccinimidyl suberate, Thermo Scientific) at complex/BS3 ratio of 1:5 (w/w) in crosslinking buffer (25 mM HEPES-NaOH, pH 7.6, 150 mM NaCl, 1 mM DTT) for 2 h on ice. The reaction was quenched by adding saturated ammonium bicarbonate solution followed by incubation on ice (45 min). Crosslinked samples were concentrated using spin concentrators (Millipore) and separated on NuPAGE 3–8% Tris-Acetate gels run in Tris-Acetate SDS running buffer (Invitrogen). Bands corresponding to crosslinked complexes were excised, crosslinked complex proteins reduced, alkylated and trypsin digested following standard procedures. Crosslinked peptides were fractionated using SCX-StageTips following published protocols for linear peptides and desalted using C18 StageTips^41^.

*Mass spectrometry.* Peptides were analysed on an LTQ Orbitrap Velos mass spectrometer coupled with an UltiMate 3000 Rapid Seperation LC system (Thermo Fisher Scientific). The column was packed into a spray emitter (75-μm inner diameter, 8-μm opening, 250-mm length; New Objectives) with C18 material (ReproSil-Pur C18-AQ 3 μm; Dr Maisch, Ammerbuch-Entringen, Germany) using an air pressure pump (Proxeon Biosystems). Mobile phase A consisted of water and 0.1% formic acid. Mobile phase B consisted of 80% acetonitrile and 0.1% formic acid. Peptides were loaded onto the column with 2% B at 500 nl min^−1^ flow rate and eluted at 300 nl min^−1^ flow rate in two steps: linear increase from 2% B to 40% B in 139 min; then increase from 40 to 95% B in 11 min. The eluted peptides were directly sprayed into the mass spectrometer. Peptides were analysed using a high/high strategy: both MS spectra and MS2 spectra were acquired in the Orbitrap. MS spectra were recorded at 100,000 resolution. The eight highest intensity peaks with a charge state of three or higher were selected in each cycle for ion trap fragmentation. Fragments were produced using CID with 35% normalized collision energy and detected by the Orbitrap at 7,500 resolution. Dynamic exclusion was set to 90s and repeat count was 1.

*Data processing*. The mass spectrometric raw files were processed into peak lists using MaxQuant (version 1.3.0.5) (ref. 41) at default parameters except for ‘top MS/MS peaks per 100 Da’ being set to 100. Search was conducted against TAF complex sequences using Xi software (version 1.3.355). Search parameters were MS accuracy, 6 p.p.m.; MS/MS accuracy, 20 p.p.m.; enzyme, trypsin; crosslinker, BS3 (including BS3 modification); max. missed cleavages, 4; fixed modification, carbamidomethylation on cysteine; variable modifications, oxidation on methionine; crosslinkable amino acids, N terminus, lysine, serine, tyrosine and threonine; fragments, b and y ions with loss of H_2_O, NH_3_ and CH_3_SOH. The data have been validated by 5% FDR with manual validation. UniProt protein accession numbers of the protein sequences used to search the database were as follows: TAF2 (Q6P1X5-1; with a sequence variation R^785^G; European Nucleotide Archive AAC68502.1), TAF4 (O00268-1), TAF5 (Q15542-1), TAF6 (P49848-1), TAF8 (Q7Z7C8-1), TAF9 (Q16594-1), TAF10 (Q12962-1), and TAF12 (Q16514-1). Crosslinks observed in artificially introduced sequences (for example, TEV cleavage sites in polyproteins or purification tags) were not included in the search. The MS data were deposited to the ProteomeXchange Consortium^66^ via the PRIDE partner repository with the data set identifier PXD001454 ( http://www.proteomexchange.org).

### Antibody production and purification

TAF2 antibodies were generated by immunizing rabbits with purified TAF2. Antibody purification was done as described^67^ with the following modifications: 2 mg of recombinant full-length human TAF2 were fixed on 400 μl Affi-Gel 10/15 beads (Bio-Rad) for 2 h at 4 °C with gentle agitation in PBS. Free active esters were blocked with 1 M ethanolamine HCl (pH 8) solution for 1 h at 4 °C under gentle agitation. The TAF2-bound gel was transferred to a column and washed four times with 10 volumes of PBS. Ten ml of rabbit polyclonal antibody sera raised against human TAF2 was applied twice and the column was washed with 10 ml of PBS before elution. Bound antibodies were eluted with 0.1 M glycine (pH 2.5) buffer. Fractions of purified antibody (500 μl) were collected and quickly neutralized by adding 50 μl 2 M Tris-HCl (pH 8.8) buffer.

### Protein extract preparations and immunprecipitation and MudPIT analyses

HeLa cell nuclear extract (NE) preparations and immunoprecipitations were done as described^68^ with minor modifications. Supernatant containing the cytoplasm was precipitated by adding stepwise 0.3 g ml^−1^ ammonium sulfate under agitation (4 °C, 30 min). Precipitated proteins were collected by centrifugation (30,000*g*, 4 °C, 20 min), resuspended and dialysed overnight.

Immunoprecipitated proteins were eluted from the protein G columns with 0.1 M glycine (pH 2.5) and quickly neutralized with 2 M Tris-HCl (pH 8.8). For MudPIT^69^ analyses, protein mixtures were trichloroacetic acid precipitated, urea denaturated, reduced, alkylated and digested with endoproteinase Lys-C followed by modified trypsin digestion. Peptide mixtures were loaded onto a triphasic 100-mm diameter fused silica microcapillary column^70^. Loaded columns were placed in-line with a Quaternary Dionex Ultimate 3000 HPLC pump and a LTQ Velos linear ion trap mass spectrometer equipped with a nano-LC electrospray ionization source (Thermo Fischer Scientific). A fully automated 12-steps MudPIT run was performed during which each full MS scan (from 300 to 1,700 *m*/*z* range) was followed by 20 MS/MS events using data-dependent acquisition^69^. Proteins were identified by database searching using SEQUEST with ThermoProteome Discoverer 1.4 (Thermo Fischer Scientific)^71^. Tandem mass spectra were searched against a human protein sequence database (from the *Homo sapiens* 2013-04-03 Swissprot release). In all searches, cysteine residues were considered to be fully carboxyamidomethylated (+57 Da statically added) and methionine to be oxidized (+16 Da dynamically added). Relative protein abundance for each protein in a given sample was estimated by normalized spectral abundance factor^72^. Normalized spectral abundance factor values were calculated from the spectral counts of each identified protein. Larger proteins tend to contribute more peptide/spectra and, therefore, spectral counts were divided by protein length to provide a spectral abundance factor (SAF). SAF values were then normalized against the sum of all SAF values in the corresponding run allowing the comparison of protein levels across different runs. The MS proteomics data were deposited to the ProteomeXchange Consortium^66^ via the PRIDE partner repository with the data set identifier PXD001427.

### Immunofluorescence

Indirect immunofluorescence tests were performed as described^30^ with the following modifications: cells were fixed with 4% paraformaldehyde for 15 min at room temperature (RT) and then permeabilized with 0.1% Triton-X100 for 20 min at RT, incubated for 1 h at RT with either an anti-TAF2 (rabbit polyclonal serum; 3038; described above; diluted 1:100)+anti-TAF8 (mouse monoclonal antibody (mAb) 1FR-1B6 (ref. 6); diluted 1:1,000) or anti-TAF2+anti-TAF10 (mAb 6TA-2B11 (ref. 6); diluted 1:1,000) antibody mix followed by incubation (RT, 1 h) with secondary antibody mix including Alexa488-labelled goat anti-rabbit mAb (Life Technologies, catalogue number A-11034, diluted 1:3,000; detects anti-TAF2) and Alexa568-labelled goat anti-mouse mAb (Life Technologies, catalogue number A-11004, diluted 1:3,000; detects either anti-TAF8 or anti-TAF10). As negative control, cells were incubated with secondary antibodies only to quantify background signal. Cells were mounted using Vectashield mounting medium with DAPI (Vector laboratories Inc.). Images were analysed on a Leica widefield fluorescence microscope (DMRXA2) equipped with a CoolSnap HQ camera ( × 63 or × 100 magnification). Fluorescence intensity measurements in the cell cytoplasm were performed using Fiji software; intensity values were normalized to background signals.

### siRNA transfection

siRNAs targeting TAF8 (ON-TARGETplus SMARTpool siRNA J-015912-20, J015912-19, J015912-18, J015912-17; Dharmacon; ThermoSientific) and non-targeting control (D-001810-10-20) were transfected into HeLa cells using Lipofectamine 2000 transfection reagent (Invitrogen). Cells were fixed for immunofluorescence experiments 48 h after transfection.

### Electron microscopy

*Specimen preparation*. 8TAF sample was stabilized by mild glutaraldehyde crosslinking (GraFix^73^). Two-hundred μl purified 8TAF complexes were loaded on a 4-ml centrifugation tube containing a 10 to 30% glycerol and a 0 to 0.15% glutaraldehyde gradient followed by centrifugation (34,000 r.p.m., 18 h, 4 °C) with a SW60 rotor (Beckman Coulter). Fractions containing stabilized sample were deposited onto a buffer exchange column (Zeba spin desalting columns, Pierce) to remove excess glycerol. Specimen was adsorbed onto a thin layer of carbon deposited on an electron microscopy grid and negatively stained for 45 s with 2% of uranyl acetate. Particles were imaged using a transmission electron microscope (Tecnai F20 G2, FEI) equipped with a field emission gun operating at 200 kV. Images were recorded under low-dose condition (total dose of 40–50 e Å^−2^) on a 2,048 × 2,048 CCD camera (Ultrascan 1000, Gatan Inc., Pleasanton) at a magnification of 50,000 resulting in a pixel spacing on the specimen of 0.21 nm.

*Random conical tilt reconstructions*. The initial reference volumes were obtained by random conical tilt using XMIPP^74^ and IMAGIC^75^ software packages. Two consecutive images of the same area were taken at 45° and 0° tilt angles under low-dose conditions. A total of 1,546 tilt pairs were selected manually using XMIPP. Untilted images were aligned using iteratively refined two-dimensional class averages as references and multivariance statistical analysis and Hierarchical Ascendant Classification for clustering into 50 class averages with IMAGIC. Fifty volumes calculated from two-dimensional classes were aligned, clustered and averaged using XMIPP MLtomo to compensate for the missing cone, resulting in five random conical tilt (RCT) reconstructions.

*Structure refinement*. The best volume was used as reference for refinement cycles using a data set of 35,145 untilted molecular images windowed with the Boxer application of the EMAN2 software package^76^ and coarsened by two resulting in a pixel spacing of 4.2 Å. Image sorting was found necessary to select the most homogeneous particles since part of the structure was flexible and prevented convergence. Sorting was performed by using first XMIPP then subsequently the RELION software package^77^. Final 3D reconstruction was performed in RELION with 2,361 sorted particles resutling in a structure with 37 Å resolution as estimated by the 0.5 Fourier Shell Correlation criteria. Images were prepared using Chimera software ( http://www.cgl.ucsf.edu/chimera).

## Author contributions

S.T., C.V., L.T. and I.B. designed and interpreted the experiments. S.T., C.V. and M.H. produced and purified all proteins and performed characterizations. S.T. determined the crystal structures. E.S. prepared the antibodies and carried out all the anti-TAF2 IPs. V.C. and M.F. performed MudPIT experiments and M.F. and L.T. analysed them. I.-O.E. and C.V.R. performed and analysed native mass spectrometry experiments. S.C. and L.T. prepared and interpreted cell-based experiments. G.P., C.S. and P.S. prepared EM grids, collected and analysed data and calculated reconstructions. J.Z. and J.R. performed and analysed CLMS experiments. S.T., L.T. and I.B. wrote the manuscript with input from all authors.

## Additional information

**Accession codes:** Atomic coordinates and structure factors are deposited in the Protein Data Bank (PDB 4WV4; 4WV6). Mass spectrometry data has been deposited in the ProteomeXchange database (PXD001454; PXD001427).

**How to cite this article:** Trowitzsch, S. *et al.* Cytoplasmic TAF2–TAF8–TAF10 complex provides evidence for nuclear holo–TFIID assembly from preformed submodules. *Nat. Commun.* 6:6011 doi: 10.1038/ncomms7011 (2015).

## Supplementary Material

Supplementary Information Supplementary Figures 1-8, Supplementary Tables 1-3, Supplementary Methods and Supplementary Reference

## Figures and Tables

**Table 1:** X-ray data collection and refinement statistics.

	**TAF8–10Native**	**TAF8–10S-SAD**	**Importin α1/TAF8–NLSNative**
*Data collection*			
Space group	P3_1_21	P3_1_21	P2_1_2_1_2_1_
Cell dimensions			
*a*, *b*, *c* (Å)	51.32, 51.32, 144.40	51.30, 51.30, 144.70	54.27, 77.72, 128.57
α, β, γ (°)	90, 90, 120	90, 90, 120	90, 90, 90
Wavelength	0.98011	1.90745	0.93340
Resolution (Å)	44.44–1.91 (1.98–1.91)*	48.23–2.61	49.54–1.75 (1.81–1.75)
*R*_merge_	2.92 (87.98)	2.00	5.00 (77.45)
*I*/*σI*	23.4 (1.68)	48.43	19.18 (2.03)
Completeness (%)	93.91 (58.53)	100.00	99.60 (99.14)
Redundancy	4.80 (4.30)	20.91	4.60 (4.60)
			
*Refinement*			
Resolution (Å)	44.44–1.91		49.54–1.75
No. of reflections	16,793 (1,630)	12,943	55,423 (5,436)
*R*_work_/*R*_free_	20.5 (33.1)/23.7 (35.5)		15.3 (23.9)/18.0 (27.2)
No. of atoms	1,474		3,877
Protein	1,404		3,366
Ligand/ion	7		48
Water	63		463
B-factors			
Protein	58.2		30.9
Ligand/ion	59.9		59.6
Water	51.9		44.8
*R.m.s. deviations*			
Bond lengths (Å)	0.003		0.008
Bond angles (°)	0.681		1.158

R.m.s., root mean squared.

^*^Values in parentheses are for highest-resolution shell.
