# Supplementary material for: Cytoplasmic TAF2–TAF8–TAF10 complex provides evidence for nuclear holo–TFIID assembly from preformed submodules
Source: Nat Commun. 2015 Jan 14;6:6011. doi: 10.1038/ncomms7011 (PMC4309443; doi:10.1038/ncomms7011)
Supplement: Supplementary Information — Supplementary Figures 1-8, Supplementary Tables 1-3, Supplementary Methods and Supplementary Reference [file ncomms7011-s1.pdf]

SUPPLEMENTARY INFORMATION

Cytoplasmic TAF2-TAF8-TAF10 complex provides evidence for nuclear holo-TFIID assembly from preformed submodules.

Simon Trowitzsch[1,2], Cristina Viola[1,2], Elisabeth Scheer[3], Sascha Conic[3], Virginie Chavant[4], Marjorie Fournier[3], Gabor Papai[5], Ima-Obong Ebong[6], Christiane Schaffitzel[1,2], Juan Zou[7], Matthias Haffke[1,2], Juri Rappsilber[7,8], Carol V. Robinson[6], Patrick Schultz[5], Laszlo Tora[3,#] & Imre Berger[1,2,9,#]

[1] European Molecular Biology Laboratory, Grenoble Outstation, 6 rue Jules Horowitz, 38042 Grenoble, France

[2] Unit for Virus Host-Cell Interactions, Univ. Grenoble Alpes-EMBL-CNRS, 6 rue Jules Horowitz, 38042 Grenoble, France.

[3] Cellular Signaling and Nuclear Dynamics Program, Institut de Génétique et de Biologie Moléculaire et Cellulaire, UMR 7104, INSERM U964, 1 rue Laurent Fries, 67404 Illkirch, France.

[4] Proteomics Platform, Institut de Génétique et de Biologie Moléculaire et Cellulaire, UMR 7104, INSERM U964, 1 rue Laurent Fries, 67404 Illkirch, France.

[5] Integrated Structural Biology Department, Institut de Génétique et de Biologie Moléculaire et Cellulaire, UMR 7104, INSERM U964, 1 rue Laurent Fries, 67404 Illkirch, France.

[6] Chemistry Research Laboratory, University of Oxford, South Parks Road, Oxford, Oxfordshire, OX1 3TA, United Kingdom.

[7] Wellcome Trust Centre for Cell Biology, University of Edinburgh, Mayfield Road, Edinburgh EH9 3JR, United Kingdom.

[8] Institute of Bioanalytics, Department of Biotechnology, Technische Universität Berlin, 13353 Berlin, Germany

[9] School of Biochemistry, Bristol University, Bristol BS8 1TD, United Kingdom

[#] Corresponding authors: Laszlo Tora (laszlo.tora@igbmc.fr) & Imre Berger (iberger@embl.fr)

Supplementary Figure 1

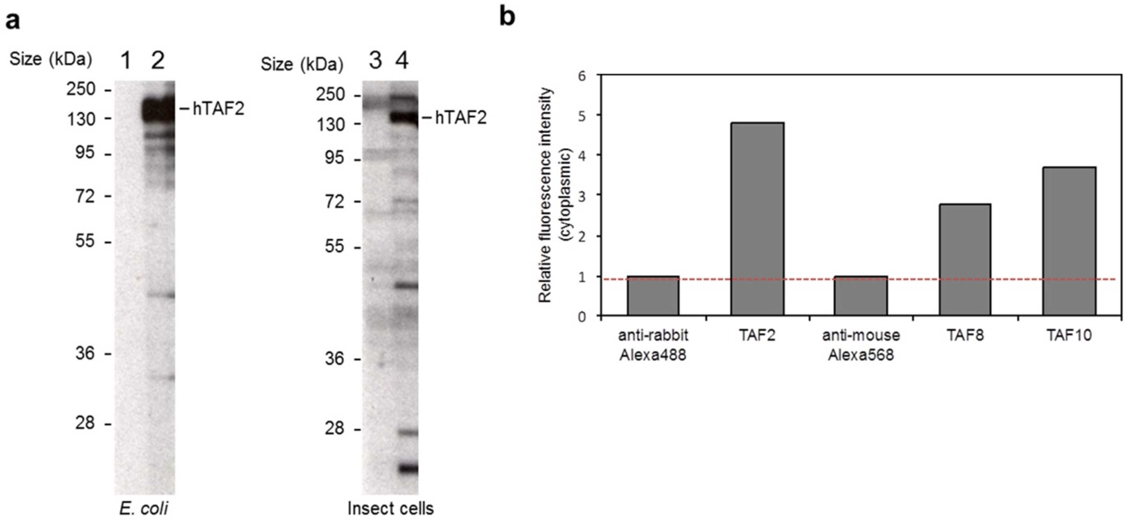

Analysis of polyclonal anti-TAF2 antibodies and immunofluorescence (IF) measurements.

(**a**) Analysis of polyclonal anti-TAF2 antibodies. Crude extracts of *E. coli* (left) or baculovirus-infected insect cells (right) expressing 6His-tagged human TAF2 were resolved on SDS-PAGE. Proteins were blotted onto nitrocellulose membranes and incubated with pre-immune serum (lanes 1 and 3) or with the non-purified anti-TAF2 serum 3038 (lanes 2 and 4) taken from rabbits, which were immunized with recombinant human TAF2 protein. Protein size markers are indicated on the left of each blot. The polyclonal antibody recognizes recombinant TAF2 from *E. coli* and baculovirus-infected insect cells. (**b**) Quantification of fluorescence intensities in the cytoplasm of HeLa cells by IF. Cytoplasmic fluorescence intensities of control cells treated only with fluorescently labeled secondary antibodies (anti-rabbit Alexa488 and anti-mouse Alexa568) were compared to cytoplasmic fluorescence intensities of cells treated with anti-TAF2 + anti-TAF8 or anti-TAF2 + anti-TAF10 primary antibodies and the same set of secondary antibodies. Fluorescence intensities were normalized to the background controls.

Supplementary Figure 2

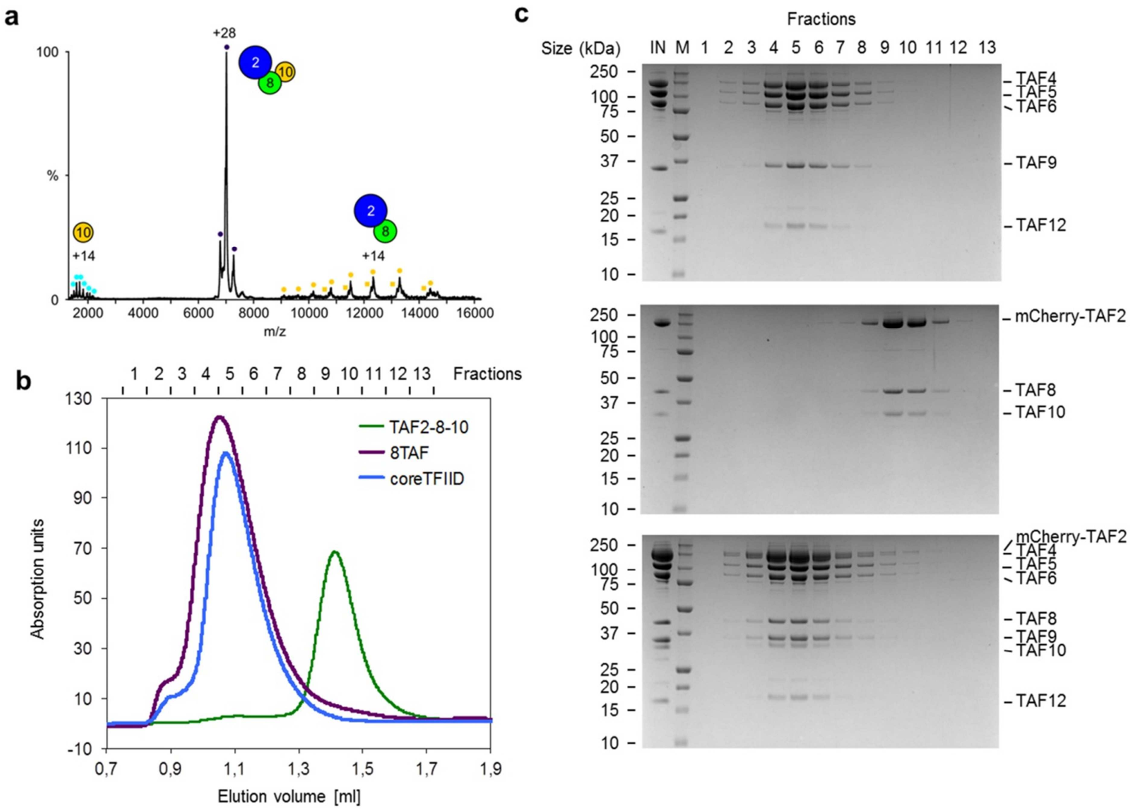

Native mass-spectrometry of a recombinant TAF2-8-10 module and incorporation of the TAF2-8-10 module into core-TFIID.

(**a**) Recombinant TAF2-8-10 complexes were electrosprayed from an aqueous ammonium acetate solution. The TAF2-8-10 module (purple dots) centers on a charge state at 7000 m/z. Charge states at around 2000 m/z (light blue dots) and 12000 m/z (yellow dots) correspond to minor amounts of TAF10 and a TAF2-8 complex, respectively. Proteins and protein complexes are schematically shown as circles. (**b** and **c**) Binding analysis of the TAF2-8-10 module with core-TFIID using size exclusion chromatography (SEC). TAF2-8-10 module, core-TFIID (TAF4, 5, 6, 9, 12) and a mixture of TAF2-8-10 module and core-TFIID were analyzed. (**b**) Elution profiles of TAF2-8-10 module (green), core-TFIID (blue) and TAF2-8-10 mixed in stoichiometric molar ratio with core-TFIID (purple) are plotted in absorption units at 280 nm versus elution volume. Fractions are numbered (top of graph). (**c**) SDS-PAGE analyses of the eluted SEC fractions are shown. Molecular masses of protein standards are indicated on the left of gel sections. Protein denominations are shown on the right. IN, input sample.

Supplementary Figure 3

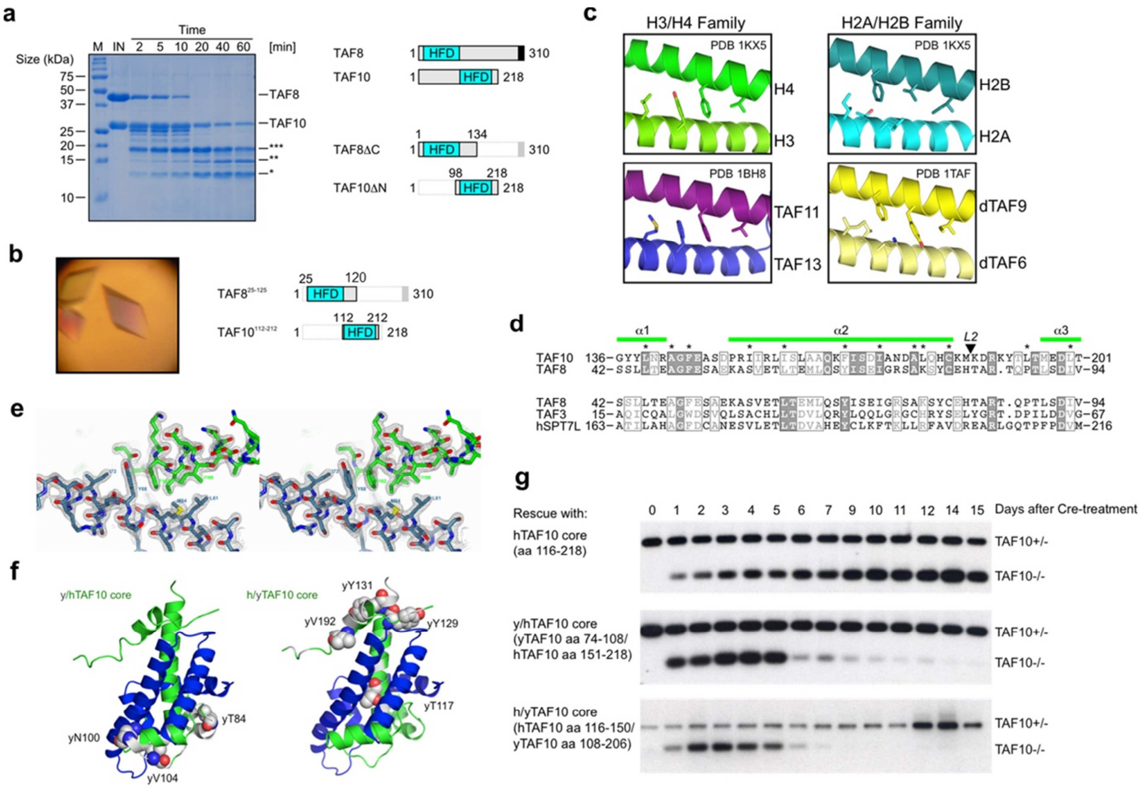

Structural analysis of a TAF8-10 complex.

(**a**) Time course of a limited proteolysis experiment with TAF8-10 using Chymotrypsin (left). Time points, protein size markers and protein identities are indicated. ***, TAF8 fragment spanning residues 1-159; **, TAF8 fragment spanning residues 1-134; *, TAF10 fragment spanning residues 98-218; IN, Input sample. HFD, histone fold domain. Bar diagrams of the proteins TAF8 and TAF10 are indicated as shown in Fig. 1c. Domain boundaries of the core TAF8-10 complex (TAF8ΔC and TAF10ΔN) are highlighted. (**b**) Image of crystals grown from a refined TAF8-10 construct (TAF8 residues 25-120 and TAF10 residues 112-212) with bar diagrams of the protein constructs. (**c**) Comparison of the central α helices of other histone fold-containing structures (PDB IDs 1KX5, 1BH8, 1TAF) showing an array of residues at the crossing of the helices. (**d**) Sequence alignment of the L1 loop regions of TAF8 and TAF10 (top). Putative L1 regions of TAF3 and human SPT7L are aligned to TAF8 (bottom). Start and end residues of the aligned sequences are indicated. Residues highlighted in Fig. 3d,e are marked by asterisks. Secondary structure elements are shown for TAF10 at the top of the alignment. Note that the L2 loop of TAF10 was removed for clarity (*L2* arrow). (**e**) Representative section of the $2F_o$-F_c electron density map (mesh) of the TAF8-10 crystal structure is shown in a stereo view, contoured at 1.5σ around the central helices of TAF8 (in blue) and TAF10 (in green). (**f**) Ribbon representations of models of the TAF8-10 complex with chimeric TAF10 molecules. The two chimeras comprise residues 74-108 of yeast and residues 151-218 of human TAF10 (left) or residues 116-150 of human and residues 108-206 of yeast TAF10 (right). Substituted yeast TAF10 residues are shown in space filling representation, colored in grey. Substituted yeast TAF10 residues which would give

rise to steric clashes, are highlighted. Color-coding is as in panel (e). (**g**) Conditional rescue experiments of TAF10$^{-/-}$ F9 embryonic carcinoma cells with TAF10 HFD and TAF10 human/yeast chimeric constructs spanning the TAF10 histone fold domain. Linearized plasmids encoding for human TAF10 (residues 116-218) and chimeric TAF10 as described in panel (f) were used to electroporate L$^-$/L2TAF10 F9 cells as described [1]. The excision of exon 2 is monitored by PCR analysis of the genomic DNA.

Supplementary Figure 4

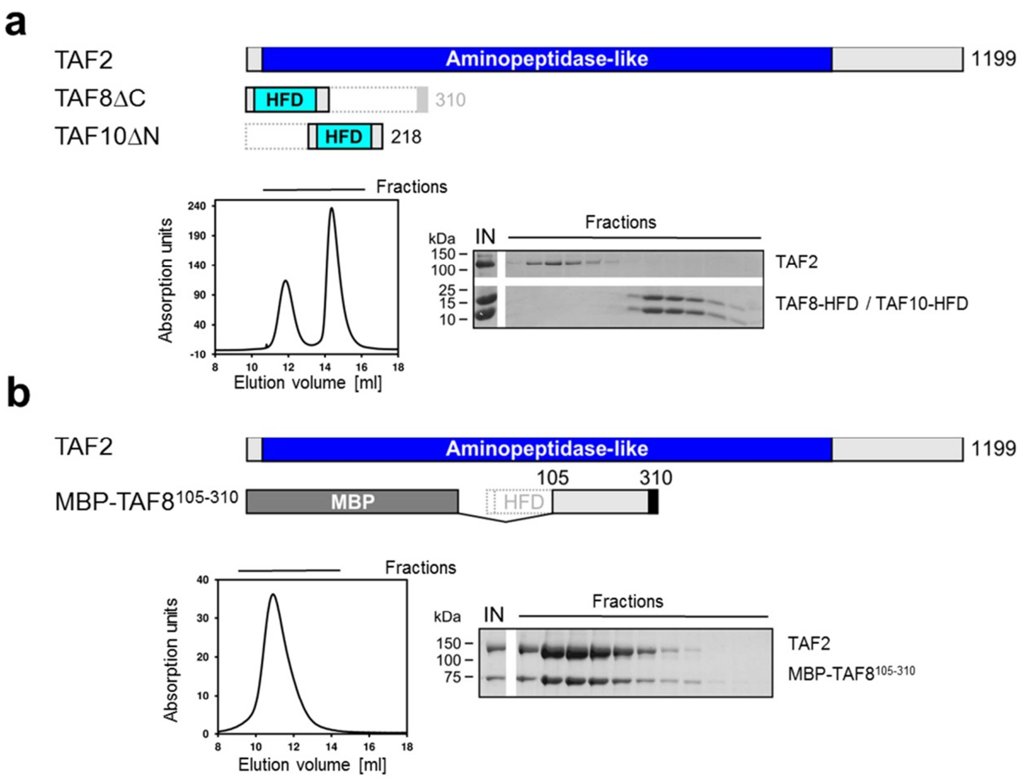

TAF2 interacts with the C-terminal region of TAF8 but not with the core complex of TAF8-10.

(**a**) Binding analysis of TAF2 with the core construct TAF8ΔC-TAF10ΔN using gel filtration. The elution profile monitored at an absorption wavelength of 280 nm versus elution volume is shown on the left and the SDS-PAGE analysis of peak fractions is shown on the right. (**b**) Similar binding experiment as in (a) but with an MBP-fusion construct of the unstructured C-terminal region of TAF8 (TAF8 residues 105-310). Protein size markers and protein identities are indicated. IN, input sample.

Supplementary Figure 5

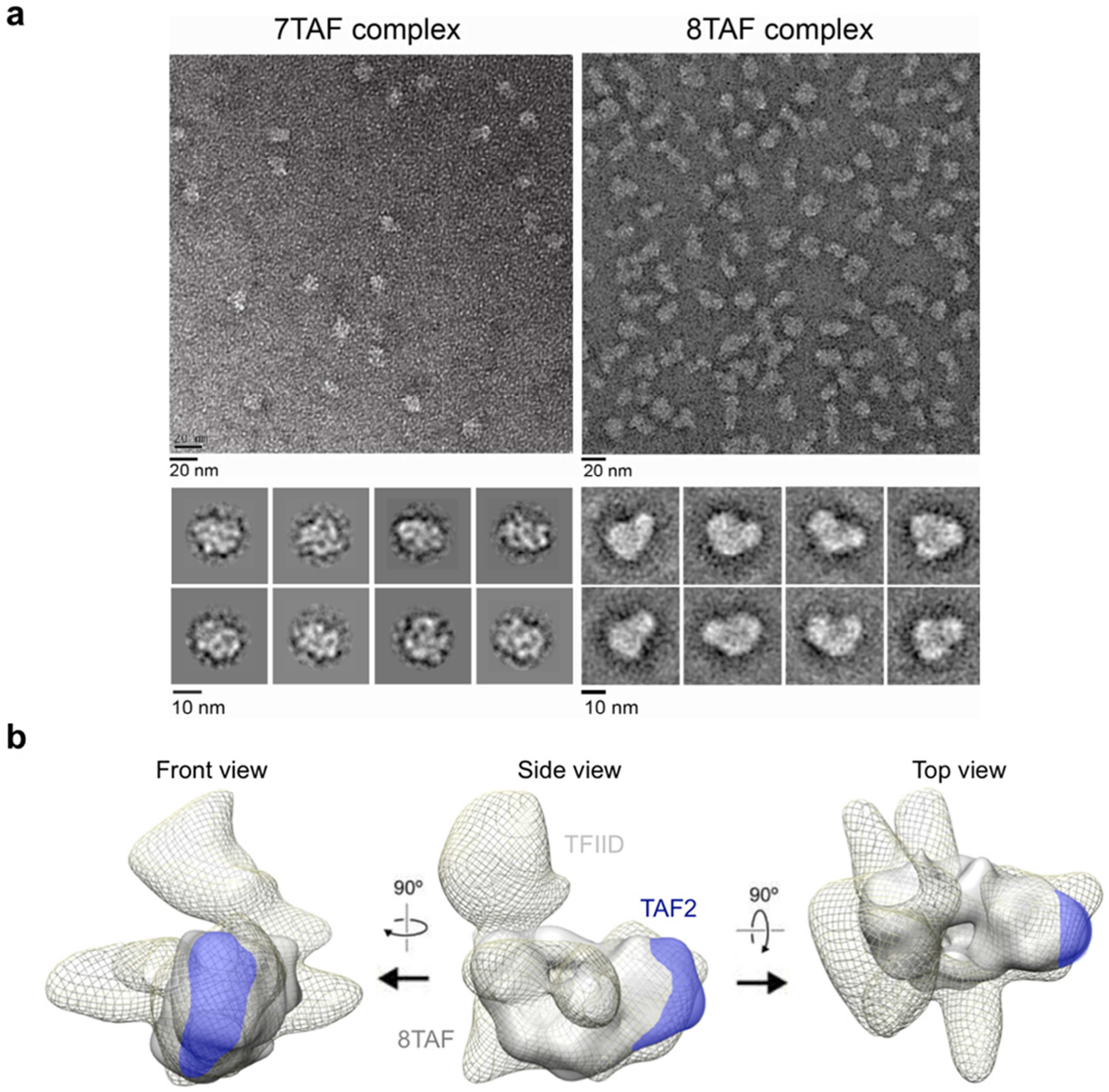

Electron microscopy of 7TAF and 8TAF complexes

(**a**) Electron micrographs and 2D class averages of 7TAF and 8TAF complexes. A section of electron micrographs from 7TAF complex consisting of TAF4, 5, 6, 8, 9, 10 and 12 is shown on the left, with representative 2D class averages shown below. A similar section from 8TAF complex comprising TAF2, 4, 5, 6, 8, 9, 10 and 12 is shown on the right, with representative 2D class averages below. Scale bars are indicated. 8TAF complex has an elongated shape as compared to more compact 7TAF complex. Additional density corresponding to TAF2 is located at one side of the 8TAF complex, adopting flexible conformations. (**b**) 3D single particle EM reconstruction of negatively stained 8TAF complex (grey) superimposed on the EM density of the holo-TFIID complex (EMD-1195, grey mesh) is shown in three views, related by a 90° rotation as indicated. Density attributed to TAF2 in the 8TAF complex is highlighted in blue.

Supplementary Figure 6

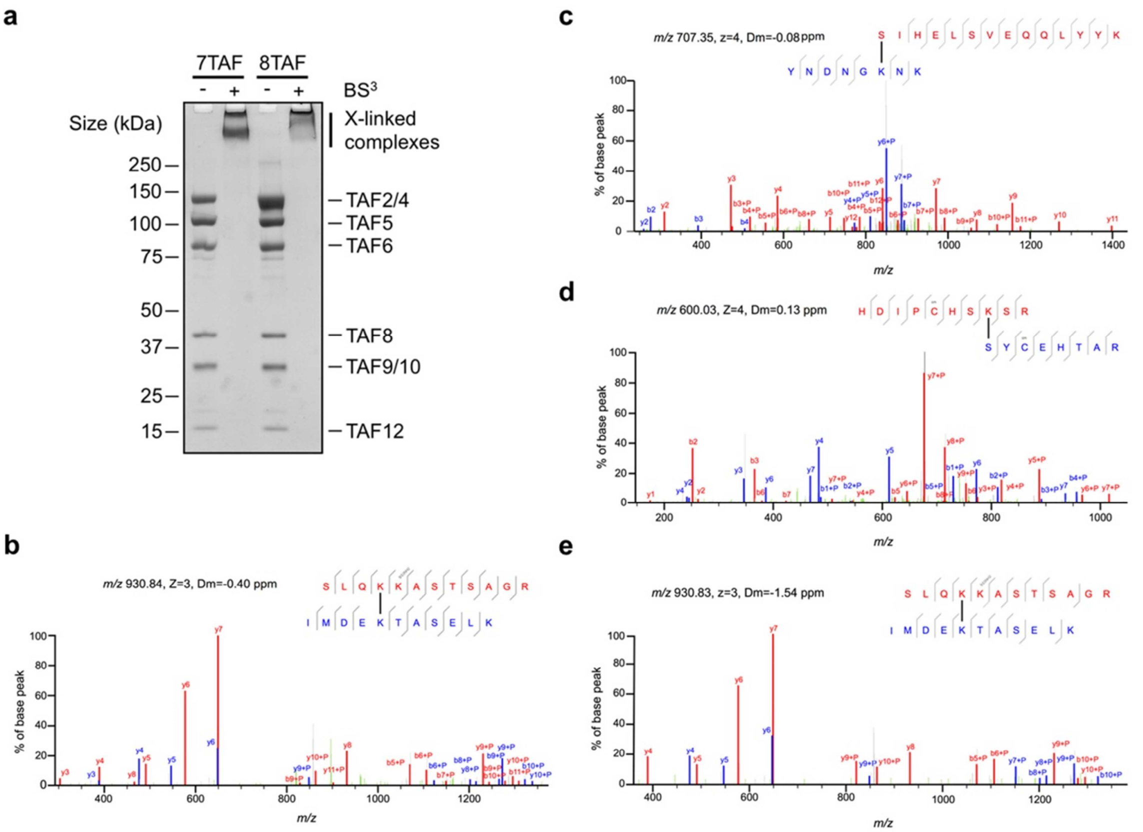

Cross-linking of 7TAF and 8TAF complexes using bifunctional crosslinker BS^3 and analysis of cross-linked peptides by mass spectrometry.

(**a**) Cross-linking efficiency of 7TAF and 8TAF complexes was assessed on NuPAGE Novex 3-8 % Tris-Acetate gels (Invitrogen). Identical amounts of 7TAF and 8TAF samples before cross-linking (-) and after cross-linking (+) were loaded on each lane. Protein size markers are shown on the left and protein identities on the right. Cross-linked complexes are indicated. (**b-e**) Representative annotated high resolution spectra of cross-linked peptides derived from 7TAF or 8TAF complexes. (**b**) Linkage TAF9 (red) K134 – TAF5 (blue) K531 observed in SLQK(xl)KASTSAGR / IMDEK(xl)TASELK (*m/z* 930,84) from 7TAF. (**c**) Linkage TAF6 (red) S212 – TAF2 (blue) K786 observed in S(xl)IHELSVEQQLYYK / YNDNGK(xl)NK (*m/z* 707,35) from 8TAF. (**d**) Linkage TAF2 (red) K595 – TAF8 (blue) S78 observed in HDIPCHSK(xl)SR / S(xl)YCEHTAR (*m/z* 600,03) from 8TAF. (**e**) Linkage TAF9 (red) K134 – TAF5 (blue) K531 observed in SLQK(xl)KASTSAGR / IMDEK(xl)TASELK (*m/z* 930,83) from 8TAF.

Supplementary Figure 7

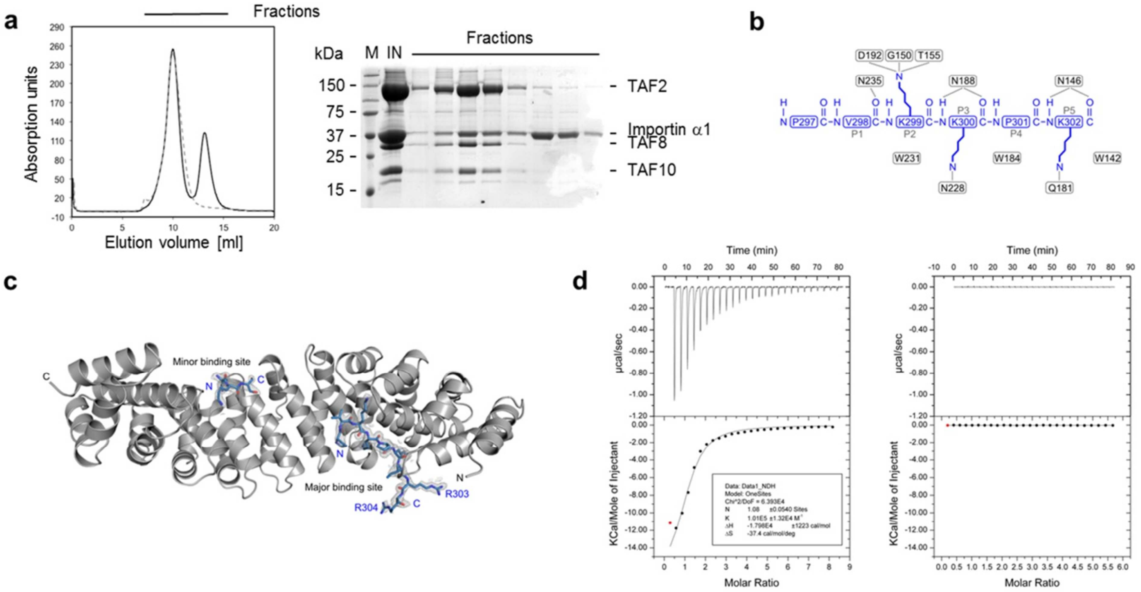

Structural and biochemical characterization of the putative nuclear import particle comprising TAF2-8-10-Importin α1.

(**a**) Binding experiment as in Supplementary Fig. 4a, but with the TAF2-8-10 complex mixed with a two-fold molar excess of Importin a1$^{\Delta IBB}$. Elution profile of the mixture is shown as a black line. The dotted line shows the elution profile of the rechromatographed material pooled from the first peak (at around 10 ml). SDS-PAGE analysis of peak fractions is shown on the right. (**b**) Schematic representation of the interactions between Importin α1 and the NLS of TAF8. Residues engaged in salt bridges, van der Waals contacts or hydrogen bondings are indicated by dashed lines. Backbone amino and carbonyl groups of the NLS peptide are schematically drawn. Residue positions are indicated. (**c**) Structure of Importin α1 with an NLS peptide of TAF8. Importin α1 molecule (grey) is shown in cartoon representation and the TAF8 peptide as sticks in blue. The $2F_o$-F_c density map contoured at 1σ around the NLS peptide fragments is shown as a grey mesh. TAF8 residues R303 and R304, which are stabilized by crystal contacts but are not engaged in Importin α1 binding, are indicated. Major and minor NLS-binding sites on the Importin α1 molecule are denoted. (**d**) TAF8-NLS peptide binding to Importin α1 assayed by isothermal titration calorimetry. The upper panel shows the added heat to the cell over time with successive additions. The excess heat added per addition was integrated from the upper panel and plotted in the lower panel as a function of the ratio of the concentration of the NLS and Importin α1 in the cell. The right panel shows a control run without Importin α1 in the cell to assess heat of dilution of the peptide.

Supplementary Figure 8

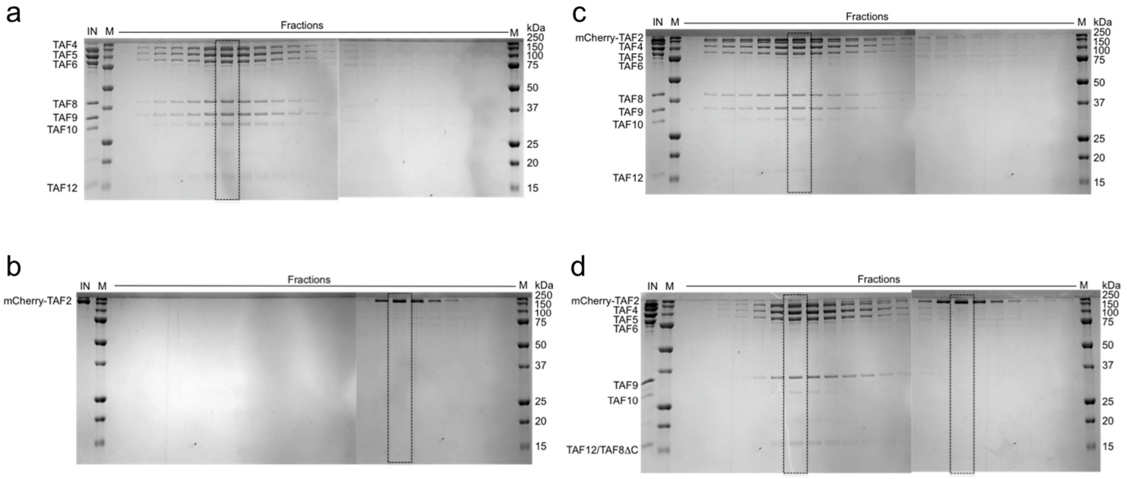

TAF8 promotes TAF2 incorporation in TFIID. Original scans of Coomassie brilliant blue-stained polyacrylamide gels. Dashed boxes indicate sections of the gels that are shown in Fig. 5b. SDS-PAGE analyses of SEC runs using 7TAF (**a**), TAF2 (**b**), 8TAF (**c**), and 7TAFΔ + TAF2 (**d**) are shown.

Supplementary Table 1

MutPIT analysis of TAF2 co-immunoprecipitated proteins from nuclear and cytoplasmic HeLa cell extracts. TFIID subunits specifically enriched in TAF2 immunoprecipitations (IPs) as compared to control IP samples are indicated by protein sequence coverage (%), unique peptides and spectral counts.

	**TAF2 IP (nuclear)**			**TAF2 IP (cytoplasmic)**		
TFIID subunit	Sequence coverage (%)	Unique peptides	Spectral counts	Sequence coverage (%)	Unique peptides	Spectral counts
TAF1	13.19	14	56			
TAF2	33.19	32	573	30.94	25	424
TAF3	4.09	3	6			
TAF4	19.63	16	169			
TAF4B	9.98	6	19			
TAF5	38.63	21	131			
TAF6	36.78	20	207			
TAF7	26.65	7	44			
TAF8	48.71	10	100	36.77	6	55
TAF9	40.53	8	45			
TAF9B	23.90	5	15			
TAF10	27.06	3	34	20.64	2	15
TAF11	30.33	5	21			
TAF12	11.08	2	2			
TAF13	16.94	2	11			
TBP	7.37	2	12			

Supplementary Table 2

Native mass-spectrometry data of TAF2-8-10 complexes.

Protein / protein complex	Measured mass [Da]*	Calculated mass [Da]
TAF8	35028	34984
TAF10	23484 / 23751 (23618 ± 134)	23613
TAF8-10	57975 / 58976 (58475 ± 501)	58579
TAF2	136364 / 137526 (136945 ± 581)	137030
TAF2-8	171548 / 172754 (172151 ± 603)	172014
TAF2-8-10	195222 / 196372 (195797 ± 575)	195609

*Two series of peaks are observed in the spectra for TAF10 and TAF2 (and therefore also complexes containing these TAFs), likely due to post-translational modification. Mass averages are provided in brackets.

Supplementary Table 3

Intermolecular BS^3 protein-protein cross-links of 7TAF and 8TAF complexes.*

Cross-linked proteins	7TAF						8TAF					
(prot1-prot2)	prot1	aa	res	prot2	aa	res	prot1	aa	res	prot2	aa	res
TAF2-TAF5							TAF2	595	K	TAF5	531	K
TAF2-TAF6							TAF2	786	K	TAF6	212	S
TAF2-TAF8							TAF2	595	K	TAF8	78	S
							TAF2	786	K	TAF8	178	K
							TAF2	1110	K	TAF8	178	K
TAF2-TAF9							TAF2	595	K	TAF9	135	K
TAF4-TAF5							TAF4	887	K	TAF5	318	K
							TAF4	888	K	TAF5	292	K
	TAF4	***945***	***K***	***TAF5***	***518***	***K***	***TAF4***	***945***	***K***	***TAF5***	***518***	***K***
	TAF4	***955***	***K***	***TAF5***	***518***	***K***	***TAF4***	***955***	***K***	***TAF5***	***518***	***K***
	TAF4	958	K	TAF5	437	K						
	TAF4	***958***	***K***	***TAF5***	***440***	***K***	***TAF4***	***958***	***K***	***TAF5***	***440***	***K***
							TAF4	971	K	TAF5	407	K
TAF4-TAF9	***TAF4***	***929***	***K***	***TAF9***	***130***	***K***	***TAF4***	***929***	***K***	***TAF9***	***130***	***K***
	TAF4	***945***	***K***	***TAF9***	***108***	***K***	***TAF4***	***945***	***K***	***TAF9***	***108***	***K***
	TAF4	***955***	***K***	***TAF9***	***108***	***K***	***TAF4***	***955***	***K***	***TAF9***	***108***	***K***
	TAF4	958	K	TAF9	135	K						
	TAF4	989	K	TAF9	108	K						
TAF4-TAF12	***TAF4***	***868***	***K***	***TAF12***	***62***	***K***	***TAF4***	***868***	***K***	***TAF12***	***62***	***K***
							TAF4	868	K	TAF12	141	K
TAF5-TAF6	***TAF5***	***318***	***K***	***TAF6***	***8***	***K***	***TAF5***	***318***	***K***	***TAF6***	***8***	***K***
	TAF5	318	K	TAF6	48	K						
							TAF5	531	K	TAF6	389	K
TAF5-TAF8	TAF5	407	K	TAF8	178	K						
	TAF5	416	K	TAF8	178	K						
	TAF5	531	K	TAF8	178	K						
TAF5-TAF9	***TAF5***	***531***	***K***	***TAF9***	***134***	***K***	***TAF5***	***531***	***K***	***TAF9***	***134***	***K***
	TAF5	***531***	***K***	***TAF9***	***135***	***K***	***TAF5***	***531***	***K***	***TAF9***	***135***	***K***
TAF6-TAF8	***TAF6***	***65***	***K***	***TAF8***	***20***	***K***	***TAF6***	***65***	***K***	***TAF8***	***20***	***K***
	TAF6	***65***	***K***	***TAF8***	***78***	***S***	***TAF6***	***65***	***K***	***TAF8***	***78***	***S***
	TAF6	65	K	TAF8	178	K						
	TAF6	***110***	***K***	***TAF8***	***20***	***K***	***TAF6***	***110***	***K***	***TAF8***	***20***	***K***
	TAF6	166	K	TAF8	178	K						
	TAF6	169	K	TAF8	178	K						
							TAF6	179	K	TAF8	178	K
	TAF6	181	K	TAF8	178	K						
							TAF6	193	S	TAF8	178	K
							TAF6	195	K	TAF8	178	K
	TAF6	***212***	***S***	***TAF8***	***178***	***K***	***TAF6***	***212***	***S***	***TAF8***	***178***	***K***
	TAF6	238	K	TAF8	178	K						
	TAF6	342	K	TAF8	178	K						
	TAF6	361	K	TAF8	178	K						
	TAF6	367	K	TAF8	178	K						
TAF6-TAF9	***TAF6***	***65***	***K***	***TAF9***	***10***	***K***	***TAF6***	***65***	***K***	***TAF9***	***10***	***K***
	TAF6	***65***	***K***	***TAF9***	***24***	***K***	***TAF6***	***65***	***K***	***TAF9***	***24***	***K***
	TAF6	***65***	***K***	***TAF9***	***135***	***K***	***TAF6***	***65***	***K***	***TAF9***	***135***	***K***
							TAF6	110	K	TAF9	10	K
TAF8-TAF9	***TAF8***	***20***	***K***	***TAF9***	***10***	***K***	***TAF8***	***20***	***K***	***TAF9***	***10***	***K***
	TAF8	***178***	***K***	***TAF9***	***135***	***K***	***TAF8***	***178***	***K***	***TAF9***	***135***	***K***
TAF9-TAF12	***TAF9***	***62***	***K***	***TAF12***	***107***	***K***	***TAF9***	***62***	***K***	***TAF12***	***107***	***K***
	TAF9	***62***	***K***	***TAF12***	***114***	***K***	***TAF9***	***62***	***K***	***TAF12***	***114***	***K***
TAF10-TAF12	TAF10	177	K	TAF12	141	K						
							TAF10	189	K	TAF12	157	K

* Mass-spectrometry was used to identify thirty-seven unique cross-links in each sample. Cross-linked residues common to 7TAF and 8TAF are highlighted (bold and italic letters). prot stands for protein; aa for amino acid number; res for residue.

Supplementary Methods

DNA constructs. Coding sequences of full-length TAF8 (Uniprot accession number Q7Z7C8) and TAF10 (Uniprot accession code Q12962) were synthesized at GenScript (New Jersey, USA) as a polyprotein construct and cloned into pPBac vector from the MultiBac suite via restriction sites *Bst*EII and *Rsr*II. The triple alanine mutant of the TAF8-10 polyprotein construct was generated by substituting the *Bst*EII-*Apa*I fragment with a synthetic DNA fragment (GenScript) carrying mutated codons for TAF8 residues 185-187 (DVE to AAA), 222-224 (PYL to AAA) and 293-295 (PYL to AAA). ORFs coding for deletion constructs of TAF10 (with engineered N-terminal, Tobacco Etch virus (TEV)-cleavable deca-histidine tag) and TAF8 were subcloned into MultiBac transfer vectors pFL and pIDC.

The TAF2 coding sequence (UniProt accession number Q6P1X5 VAR_027855) was cloned into a modified pFL vector coding for an engineered N-terminal TEV-cleavable deca-His tag via restriction sites *Sal*I and *Hind*III. The mCherry-TAF2 construct was cloned by inserting the mCherry-coding sequence via the *Sal*I cleavage site into the pFL-HisTEVTAF2 vector. The MBP-TAF2 construct was generated in analogy to the mCherry-TAF2 construct. Transfer vectors were either first fused *in vitro* by Cre-LoxP recombination or directly integrated into the EmBacY baculovirus genome by *in vivo* Tn7 transposition using standard protocols.

For MBP-fusion constructs, coding sequences of truncation versions of the TAF8 protein were amplified via PCR from the synthetic polyprotein construct and cloned into pMAL-c vector (Novagen) with engineered C-terminal hexa-histidine tags via SLIC.

Protein Production and Purification. *E. coli* Rosetta™(DE3) cells (Novagen) were transformed with plasmids pMAL-c_TAF8_105-310, pMAL-c_TAF8_105-260, pMAL-c_TAF8_141-310, and pMAL-c_TAF8_200-310. Cells were grown in LB broth (Miller's) medium supplemented with 34 µg ml^{-1} Chloramphenicol and 100 µg ml^{-1} Ampicillin at 37 °C. Temperature was decreased to 20 °C at an optical density (OD_{600}) of 0.4. Protein production was induced at an OD_{600} of 0.8 by addition of

isopropyl β-D-1-thiogalactopyranoside (IPTG) to a final concentration of 1 µM. Cells were harvested 18 h post induction by centrifugation. Cell pellets were flash frozen in liquid nitrogen and resuspended in lysis buffer (50 mM HEPES-NaOH, pH 7.6, 300 mM NaCl, 5 mM Imidazole, 1 µM Leupeptin, 1 µM Pepstatin, 50 µg ml^{-1} lysozyme) supplemented with 1 tablet of cOmplete EDTA-free protease inhibitor cocktail (Roche). Crude extracts were prepared by sonication and cleared by centrifugation (Beckman JA-20 rotor, 45 min, 20000 rpm, 4 °C). All purification steps were performed at 4 °C on ÄKTA prime and purifier systems (GE Healthcare). Soluble extracts were passed over a 5 ml column of TALON® metal affinity resin (Clontech) equilibrated in lysis buffer. The resin was washed with 10 cv of washing buffer (50 mM HEPES-NaOH, pH 7.6, 300 mM NaCl, 5 mM Imidazole, 0.01% [v/v] NP-40, 1 µM Leupeptin, 1 µM Pepstatin) and 10 cv of washing buffer without NP-40. MBP-fusions were eluted by a linear imidazole gradient of 16 cv into elution buffer (50 mM HEPES-NaOH, pH7.6, 300 mM NaCl, 300 mM Imidazole, 1 µM Leupeptin, 1 µM Pepstatin). Peak fractions were pooled and concentrated to 1 ml via ultrafiltration in 15 ml, 10 MWCO spin concentrators (Millipore). Fusion proteins were further purified to homogeneity via size exclusion chromatography on a Superdex 200 16/60 column (GE Healthcare) in a buffer comprising 25 mM HEPES-NaOH, pH 7.6, 300 mM NaCl and supplemented with cOmplete EDTA-free protease inhibitor cocktail (Roche). Proteins were concentrated to ~ 10 mg ml^{-1}, flash-frozen in liquid nitrogen and stored at – 80 °C in aliquots.

Pellets of baculovirus-infected Sf21 cells expressing TAF2 constructs were resuspended in lysis buffer (50 mM HEPES-NaOH, pH 7.5, 500 mM NaCl, 10 mM Imidazole, 1 µM Leupeptin, 1 µM Pepstatin, supplemented with cOmplete EDTA-free protease inhibitor cocktail) and crude extracts were prepared by sonication and subsequently cleared by centrifugation (Beckman JA-25.50 rotor, 1 h, 25000 rpm, 4 °C). TAF2 constructs were captured from soluble extract via TALON® metal affinity resin (Clontech) in batch. The resin was extensively washed with lysis buffer and TAF2 constructs were eluted in lysis buffer supplemented with 250 mM Imidazole. Proteins TAF2 and mCherry-TAF2 were furthermore purified by ion exchange chromatography using a 5 ml SP-Sepharose HiTrap column (GE Healthcare) and eluted from the column by a linear salt gradient in a buffer comprising 25 mM HEPES-NaOH, pH 7.5, 150 to 1000 mM NaCl, 1 mM □-mercaptoethanol, 1 µM Leupeptin and 1 µM Pepstatin. Prior to ion exchange chromatography the His-tag

was removed by incubating the proteins with Tobacco Etch Virus (TEV) protease (produced in house). TAF2 constructs were finally polished by gel filtration using Superdex200 10/300 or Superose6 10/300 columns (GE Healthcare) in a buffer comprising 25 mM HEPES-NaOH, pH 7.5, 500 mM NaCl, 1 mM β-mercaptoethanol. Proteins were concentrated to ~ 5-20 mg ml^{-1}, flash-frozen in liquid nitrogen and stored at – 80 °C in aliquots.

TAF8-10 constructs were produced and purified essentially as described above for the TAF2 constructs with the exception that the lysis and SEC buffer contained 150 mM NaCl instead of 500 mM, Furthermore, protein complexes were subjected to SEC immediately after elution from the TALON resin. For crystallization purposes, the complexes were subjected to SEC in a buffer comprising 10 mM Tris-HCl, pH 7.5, 150 mM NaCl, 1 mM dithiothreitol (DTT).

Supplementary Reference

[1] Kouskouti, A., Scheer, E., Staub, A., Tora, L. & Talianidis, I. Gene-specific modulation of TAF10 function by SET9-mediated methylation. *Mol. Cell* 14, 175-182 (2004).
